# Multi-Gene Phylogeny and Morphology Reveal *Haplohelminthosporium* gen. nov. and *Helminthosporiella* gen. nov. Associated with Palms in Thailand and A Checklist for *Helminthosporium* Reported Worldwide

**DOI:** 10.3390/life11050454

**Published:** 2021-05-19

**Authors:** Sirinapa Konta, Kevin D. Hyde, Samantha C. Karunarathna, Ausana Mapook, Chanokned Senwanna, Lucas A. P. Dauner, Chandrika M. Nanayakkara, Jianchu Xu, Saowaluck Tibpromma, Saisamorn Lumyong

**Affiliations:** 1 CAS Key Laboratory for Plant Diversity and Biogeography of East Asia, Kunming Institute of Botany, Chinese Academy of Sciences, Kunming 650201, China; sirinapakonta@gmail.com (S.K.); kdhyde3@gmail.com (K.D.H.); samantha@mail.kib.ac.cn (S.C.K.); luke.dauner1@gmail.com (L.A.P.D.); jxu@mail.kib.ac.cn (J.X.); 2 Center of Excellence in Fungal Research, Mae Fah Luang University, Chiang Rai 57100, Thailand; phung.ausana@gmail.com; 3 School of Science, Mae Fah Luang University, Chiang Rai 57100, Thailand; 4 Department of Entomology and Plant Pathology, Faculty of Agriculture, Chiang Mai University, Chiang Mai 50200, Thailand; chanokned.swn@gmail.com; 5 Department of Plant Sciences, University of Colombo, Colombo 00300, Sri Lanka; chandi@pts.cmb.ac.lk; 6 Research Center of Microbial Diversity and Sustainable Utilization, Faculty of Science, Chiang Mai University, Chiang Mai 50200, Thailand; 7 Academy of Science, The Royal Society of Thailand, Bangkok 10300, Thailand

**Keywords:** 4 new taxa, Massarinaceae, morphology, multi-genes, palm fungi, Thailand

## Abstract

Palms (Arecaceae) are substrates for a highly diverse range of fungi. Many species are known as saprobes and many are important plant pathogens. Over the course of our studies of micro-fungi from palms in Thailand, two new taxa were discovered. Morphological characteristics and phylogenetic analyses of combined ITS, LSU, SSU, and *tef1-α* sequence data revealed their taxonomic positions within Massarinaceae. There are currently ten genera identified and accepted in Massarinaceae, with the addition of the two new genera of *Haplohelminthosporium* and *Helminthosporiella*, that are introduced in this paper. Each new genus is provided with a full description and notes, and each new taxon is provided with an illustration for the holotype. A list of identified and accepted species of *Helminthosporium* with morphology, host information, locality, sequence data, and related references of *Helminthosporium* reported worldwide is provided based on records in Species Fungorum 2021. This work provides a micro-fungi database of *Haplohelminthosporium*, *Helminthosporiella*, and *Helminthosporium* which can be modified and validated as new data come to light.

## 1. Introduction

In Thailand, a large number of novel fungi from a variety of hosts have been recently described, adding to the region’s highly known fungal diversity [1,2]. This diversity is supported by various factors, including host–plant species relationships, geography, seasons, air humidity, and temperature. Many interesting fungi from Thai monocotyledons such as bamboo (Poaceae) and Pandanaceae have been described in previous studies, and some new taxa and records of microfungi on palms have been published, especially from the southern region of Thailand [3,4,5,6,7,8,9,10,11]. However, more research on fungal diversity on palms in Thailand is needed.

Pleosporales is the largest order in Dothideomycetes [12] with 566 genera in 91 families accepted, while 48 genera have been placed in Pleosporales genera *incertae sedis* with an estimated stem age of 205 MYA [12,13]. Massarinaceae is a family within Pleosporales introduced by Munk [14] to accommodate the genus *Massarina*, with *M. eburnea* being designated as the type species and described based on the sexual morph [15]. Hongsanan et al. [12] and Wijayawardene et al. [13] accepted nine genera in Massarinaceae (*Byssothecium*, *Helminthosporiella*, *Helminthosporium*, *Massarina*, *Pseudodidymosphaeria*, *Pseudosplanchnonema*, *Semifissispora*, *Stagonospora*, and *Suttonomyces*).

*Helminthosporium* has the asexual morph of *H. velutinum* as the type species. It is characterized by terminal and intercalary conidiogenous cells as well as solitary conidia with distosepta [16]. The members of this genus are commonly found as saprobes and endophytes, but they are often isolated from dead corticated twigs or wood, living leaves, and soils [17,18,19,20,21,22,23]. Most *Helminthosporium* species have been described based on their asexual morph, and only a few species have been described based on both morphs viz., *H. massarinum*, *H. microsorum*, *H. oligosporum*, *H. quercicola*, *H. quercinum*, and *H. tiliae* [19,21,24]. Several species in the *Helminthosporium* complex are polyphyletic and have been placed in other genera viz. *Bipolaris*, *Curvularia*, and *Exserohilum* within Pleosporales, other families viz. Corynesporaceae, Massarinaceae, and Mycosphaerellaceae within Dothideomycetes, or other unrelated Ascomycetes groups that were initially based on morphological characteristics and later on molecular data, although some species still remain unresolved [20,25,26,27,28,29,30,31,32,33,34,35,36,37]. Wijayawardene et al. [13] approximated the number of taxa in *Helminthosporium* at 416 species. However, this genus was not updated with the DNA sequencesin the most recent monograph.

Few previous studies have investigated the *Helminthosporium*-like taxa from plants, particularly palms, in Thailand. In this study, we were able to isolate *Helminthosporium*-like taxa from palms collected in Thailand. Morphology and multi-gene phylogenetic analyses showed two *Helminthosporium*-like taxa are novel in Massarinaceae. In addition, we provide a checklist of *Helminthosporium* and the name for *Helminthosporiella stilbacea* is also validated.

## 2. Materials and Methods

### 2.1. Collection, Isolation, and Identification

The plant materials containing the fungal structures were collected from Krabi and Prachuap Khiri Khan Provinces, Thailand, from living and dead parts of palm trees (*Calamus* sp. and *Cocos nucifera*). Samples were taken to the laboratory for morphological study following the methods provided by Konta et al. [9]. Single spore isolates were obtained following the method of Senanayake et al. [38]. Measurements were taken using an Image Framework program. Illustrations were made in Adobe Photoshop CS6. Specimens and cultures were deposited in the herbarium of Mae Fah Luang University (MFLU) and Mae Fah Luang Culture Collection (MFLUCC). Faces of Fungi and Index Fungorum numbers were registered as outlined in Jayasiri et al. [39] and Index Fungorum [40], respectively.

### 2.2. DNA Extraction and Amplification (PCR)

DNA extraction was performed using the Biospin Fungus genomic DNA extraction kit-BSC14S1 (Bioflux, P.R. China) according to Dissanayake et al. [41]. Partial nucleotide genes were subjected to PCR amplification and sequencing of the large subunit (28S, LSU) [42], the internal transcribed spacer (ITS) [43], the small subunit (18S, SSU) [43], and the translation elongation factor 1-alpha (*tef1-α*) was performed [44,45]. For primers and conditions, see Table 1. PCR amplification and sequencing were carried out following Konta et al. [9]. The resulting fragments were sequenced in both forward and reverse directions, the generated DNA sequences were analysed, and the consensus sequences were computed using SeqMan software. New sequences generated in this study were deposited in GenBank (Table 2).

### 2.3. Phylogenetic Analyses

**Figure 1:**
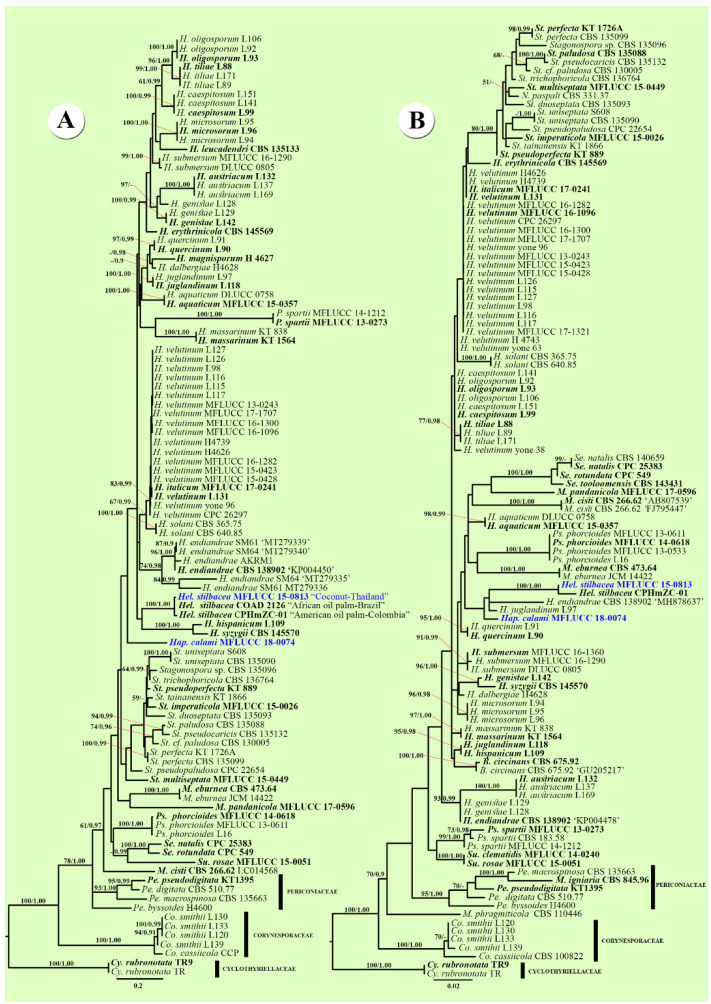
Comparison of the topology of Maximum likelihood majority rule consensus tree for the analyses of some selected Corynesporaceae, Massarinaceae, and Perioconiaceae isolates. (**A**) Phylogenetic tree of the dataset for ITS sequence data. (**B**) Phylogenetic tree of the dataset for LSU sequence data. Bootstrap support values for maximum likelihood (ML) equal to or higher than 50%, and Bayesian Posterior Probabilities (BYPP) equal to or greater than 0.90 are given above each branch. Novel taxa are in blue. Ex-type strains are in bold. The tree is rooted to *Cyclothyriella rubronotata* strains TR, TR9 (Cyclothyriellaceae).

**Figure 2:**
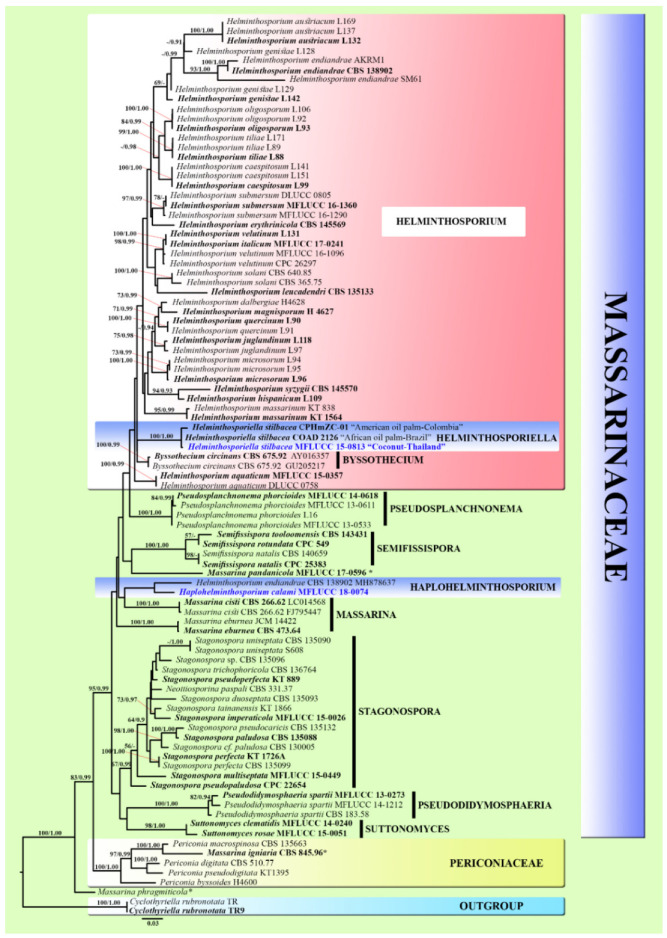
Maximum likelihood majority rule consensus tree for the analyses of Massarinaceae and sister family Perioconiaceae isolates based on a dataset of combined ITS, LSU, SSU, and *tef1-α* sequence data. Bootstrap support values for maximum likelihood (ML) equal to or higher than 50%, and Bayesian posterior probabilities (BYPP) equal to or greater than 0.90 are given above each branch. Novel taxa are in blue. Ex-type strains are in bold. The tree is rooted to *Cyclothyriella rubronotata* strains TR, TR9 (Cyclothyriellaceae).

The sequences generated in this study were subjected to a BLAST search in GenBank to identify closely related sequences. Sequence data retrieved from GenBank and recent publications were used as references [24]. Sequence data for the ITS, LSU, SSU, and *tef1-α* regions were analysed both individually and in combination. A total of 93 taxa were used for the combined phylogenetic analyses (ITS, LSU, SSU, and *tef1-α*) in order to find a natural classification placement. In addition, 103 taxa of ITS and 113 taxa of LSU were used for phylogenetic analyses. For both the individual and combined phylogenetic analyses, *Cyclothyriella rubronotata* (Cyclothyriellaceae) was selected as the outgroup taxon. Absent sequence data (i.e., ITS, LSU, SSU, *tef1-α* sequence data) in the alignments were treated with gaps as missing data. Sequence alignments were carried out with MAFFT v.6.864b [46] and were manually improved where necessary. The single gene datasets were combined using Mega7 [47]. Data were converted from fasta to nexus and PHYLIP format with Alignment Transformation Environment online, https://sing.ei.uvigo.es/ALTER/ (accessed on 15 July 2020) [48]. The tree topologies obtained from single gene sequence data were compared prior to the combined gene analysis in order to check for incongruence in the overall topology of the phylogenetic tree. Maximum likelihood (ML) analysis was accomplished using RAxML-HPC2 (v.8.2.12) on XSEDE in the CIPRES Science Gateway platform (http://www.phylo.org) (accessed on 12 May 2020) [49] with GTRGAMMA model and set as 1000 bootstrap replicates. Bayesian analysis was performed at CIPRES using Bayesian analysis on XSEDE (v.3.2.7) as part of the “MrBayes on XSEDE” tool [49,50,51]. GTR+I+G model was selected by using MrModelTest 2.2 [52] under the Akaike information criterion (AIC) as the best-fit models of the combined dataset for maximum likelihood and Bayesian analysis [52]. Bayesian posterior probabilities (BYPP) were determined by Markov Chain Monte Carlo sampling (MCMC) in MrBayes on XSEDE v.3.2.7. Six simultaneous Markov chains were run for 5,000,000 generations and trees were sampled every 1000th generation. An MCMC heated chain was set with a “temperature” value of 0.20. All sampled topologies beneath the asymptote (25%) were discarded as part of a burn-in procedure; the remaining trees (7502) were used for calculating posterior probabilities in the majority rule consensus tree. Bootstrap support values for ML and BYPP are given near to each node (Figure 1 and Figure 2). The phylogenetic trees were configured in FigTree v1.4.0 [53] and edited using Microsoft Office PowerPoint 2016 and Adobe Photoshop CS6 (Adobe Systems, San Jose, CA, USA).

**Table 2:** Taxa names, strain numbers and GenBank accession numbers of the sequences used in phylogenetic analyses.

Family	Species	Strain No.	GenBank Accession No.				References
			ITS	LSU	SSU	*tef1-α*	
Corynesporaceae	*Corynespora cassiicola*	CBS 100,822	-	GU301808	GU296144	GU349052	[54]
Corynesporaceae	*Corynespora cassiicola*	CCP	KF810854	-	GU296145	-	[54,55]
Corynesporaceae	*Corynespora smithii*	CBS 139,925	KY984299	KY984299	-	-	[21]
Corynesporaceae	*Corynespora smithii*	L120	KY984297	KY984297	-	KY984435	[21]
Corynesporaceae	*Corynespora smithii*	L130	KY984298	KY984298	KY984419	KY984436	[21]
Corynesporaceae	*Corynespora smithii*	L139	KY984300	KY984300	-	-	[21]
Cyclothyriellaceae	*Cyclothyriella rubronotata*	TR	KX650541	KX650541	-	KX650516	[56]
Cyclothyriellaceae	*Cyclothyriella rubronotata*	TR9 *	KX650544	KX650544	KX650507	KX650519	[56]
Massariaceae	*Byssothecium circinans*	CBS 675.92	-	GU205217	GU205235	GU349061	[54]
Massarinaceae	*Byssothecium circinans*	CBS 675.92	-	AY016357	AY016339	-	[57,58]
Massarinaceae	*Haplohelminthosporium calami*	MFLUCC 18-0074 *	MT928158	MT928156	MT928160	-	This study
Massarinaceae	*Helminthosporium aquaticum*	MFLUCC 15-0357	KU697302	KU697306	KU697310	-	[20]
Massarinaceae	*Helminthosporium aquaticum*	DLUCC 0758	MG098779	MG098786	MG098795	MG98585	[24]
Massarinaceae	*Helminthosporium austriacum*	L132 *	KY984301	KY984301	KY984420	KY984437	[21]
Massarinaceae	*Helminthosporium austriacum*	L169	KY984303	KY984303	-	KY984439	[21]
Massarinaceae	*Helminthosporium austriacum*	L137	KY984302	KY984302	-	KY984438	[21]
Massarinaceae	*Helminthosporium caespitosum*	L99 *	JQ044429	JQ044448	KY984421	KY984440	[21]
Massarinaceae	*Helminthosporium caespitosum*	L141	KY984305	KY984305	-	-	[21]
Massarinaceae	*Helminthosporium caespitosum*	L151	KY984306	KY984306	-	-	[21]
Massarinaceae	*Helminthosporium dalbergiae*	H 4628	LC014555	AB807521	AB797231	AB808497	[19]
Massarinaceae	*Helminthosporium endiandrae*	CBS 138902 *	KP004450	KP004478	-	-	[59]
Massarinaceae	*Helminthosporium endiandrae*	CBS 138,902	-	MH878637	-	-	[60]
Massarinaceae	*Helminthosporium endiandrae*	SM64	MT279335	-	-	-	Unpublished
Massarinaceae	*Helminthosporium endiandrae*	SM61	MT279339	-	-	-	Unpublished
Massarinaceae	*Helminthosporium endiandrae*	SM64	MT279340	-	-	-	Unpublished
Massarinaceae	*Helminthosporium endiandrae*	SM61	MT279336	-	-	-	Unpublished
Massarinaceae	*Helminthosporium endiandrae*	AKRM1	MN880136	-	-	-	Unpublished
Massarinaceae	*Helminthosporium erythrinicola*	CBS 145,569	MK876391	MK876432	-	-	[22]
Massarinaceae	*Helminthosporium genistae*	L128	KY984308	KY984308	KY984422	-	[21]
Massarinaceae	*Helminthosporium genistae*	L129	KY984309	KY984309	KY984423	-	[21]
Massarinaceae	*Helminthosporium genistae*	L142 *	KY984310	KY984310	-	-	[21]
Massarinaceae	*Helminthosporium hispanicum*	L109 *	KY984318	KY984318	KY984424	KY984441	[21]
Massarinaceae	*Helminthosporium italicum*	MFLUCC 17-0241	KY797638	KY815015	-	KY815021	[61]
Massarinaceae	*Helminthosporium juglandinum*	L97	KY984322	KY984322	KY984425	KY984445	[21]
Massarinaceae	*Helminthosporium juglandinum*	L118 *	KY984321	KY984321	-	KY984444	[21]
Massarinaceae	*Helminthosporium leucadendri*	CBS 135133 *	KF251150	KF251654	-	KF253110	[62]
Massarinaceae	*Helminthosporium magnisporum*	H 4627 *	AB811452	AB807522	AB797232	AB808498	[19]
Massarinaceae	*Helminthosporium massarinum*	KT 1564 *	AB809629	AB807524	AB797234	AB808500	[19]
Massarinaceae	*Helminthosporium massarinum*	KT 838	AB809628	AB807523	AB797233	AB808499	[19]
Massarinaceae	*Helminthosporium microsorum*	L94	KY984327	KY984327	KY984426	KY984446	[21]
Massarinaceae	*Helminthosporium microsorum*	L95	KY984328	KY984328	-	KY984447	[21]
Massarinaceae	*Helminthosporium microsorum*	L96 *	KY984329	KY984329	KY984427	KY984448	[21]
Massarinaceae	*Helminthosporium oligosporum*	L92	KY984332	KY984332	KY984428	KY984450	[21]
Massarinaceae	*Helminthosporium oligosporum*	L93 *	KY984333	KY984333	-	KY984451	[21]
Massarinaceae	*Helminthosporium oligosporum*	L106	KY984330	KY984330	-	KY984449	[21]
Massarinaceae	*Helminthosporium quercinum*	L90 *	KY984339	KY984339	KY984429	KY984453	[21]
Massarinaceae	*Helminthosporium quercinum*	L91	KY984340	KY984340	-	KY984454	[21]
Massarinaceae	*Helminthosporium solani*	CBS 365.75	KY984341	KY984341	KY984430	KY984455	[21]
Massarinaceae	*Helminthosporium solani*	CBS 640.85	KY984342	KY984342	-	-	[21]
Massarinaceae	*Helminthosporiella stilbacea*	CPHmZC-01	KX228298	KX228355	-	-	[63]
Massarinaceae	*Helminthosporiella stilbacea*	COAD 2126	MG668862	-	-	-	[64]
Massarinaceae	*Helminthosporiella stilbacea*	MFLUCC 15-0813 *	MT928159	MT928157	MT928161	MT928151	This study
Massarinaceae	*Helminthosporium submersum*	MFLUCC 16-1360 *	-	MG098787	MG098796	MG098586	[24]
Massarinaceae	*Helminthosporium submersum*	MFLUCC 16-1290	MG098780	MG098788	MG098797	MG098587	[24]
Massarinaceae	*Helminthosporium submersum*	DLUCC 0805	MG098781	MG098789	MG098798	-	[24]
Massarinaceae	*Helminthosporium syzygii*	CBS 145,570 *	MK876392	MK876433	-	-	[22]
Massarinaceae	*Helminthosporium tiliae*	L88 *	KY984345	KY984345	KY984431	KY984457	[21]
Massarinaceae	*Helminthosporium tiliae*	L89	KY984346	KY984346	-	-	[21]
Massarinaceae	*Helminthosporium tiliae*	L171	KY984343	KY984343	-	KY984456	[21]
Massarinaceae	*Helminthosporium velutinum*	yone 38	-	AB807527	AB797237	AB808502	[19]
Massarinaceae	*Helminthosporium velutinum*	yone 63	-	AB807528	AB797238	AB808503	[19]
Massarinaceae	*Helminthosporium velutinum*	MFLUCC 15-0423	KU697300	KU697304	KU697308	-	[20]
Massarinaceae	*Helminthosporium velutinum*	MFLUCC 15-0428	KU697299	KU697303	KU697307	-	[20]
Massarinaceae	*Helminthosporium velutinum*	H 4626	LC014556	AB807530	AB797240	AB808505	[19]
Massarinaceae	*Helminthosporium velutinum*	L117	KY984349	KY984349	-	KY984460	[21]
Massarinaceae	*Helminthosporium velutinum*	L126	KY984350	KY984350	-	KY984461	[21]
Massarinaceae	*Helminthosporium velutinum*	L131 *	KY984352	KY984352	KY984432	KY984463	[21]
Massarinaceae	*Helminthosporium velutinum*	CPC 26297= CBS 141,504	KX306757	KX306785	-	-	[65]
Massarinaceae	*Helminthosporium velutinum*	yone 96	LC014558	AB807529	AB797239	AB808504	[19]
Massarinaceae	*Helminthosporium velutinum*	H 4739	LC014557	AB807525	AB797235	AB808501	[19]
Massarinaceae	*Helminthosporium velutinum*	L115	KY984347	KY984347	-	KY984458	[21]
Massarinaceae	*Helminthosporium velutinum*	L116	KY984348	KY984348	-	KY984459	[21]
Massarinaceae	*Helminthosporium velutinum*	L127	KY984351	KY984351	-	KY984462	[21]
Massarinaceae	*Helminthosporium velutinum*	L98	KY984359	KY984359	KY984433	KY984466	[21]
Massarinaceae	*Helminthosporium velutinum*	H 4743	-	AB807526	AB797236	-	[19]
Massarinaceae	*Helminthosporium velutinum*	MFLUCC 16-1096	MG098783	MG098791	MG098799	MG098588	[24]
Massarinaceae	*Helminthosporium velutinum*	MFLUCC 16-1282	MG098784	MG098792	MG098800	MG098589	[24]
Massarinaceae	*Helminthosporium velutinum*	MFLUCC 17-1707	MG098785	MG098793	MG098801	MG098590	[24]
Massarinaceae	*Helminthosporium velutinum*	MFLUCC 17-1321	-	MG098794	MG098802	MG098591	[24]
Massarinaceae	*Helminthosporium velutinum*	S-076	KU697301	KU697305	KU697309	-	[20]
Massarinaceae	*Helminthosporium velutinum*	MFLUCC 15-0243	KU697301	KU697305	KU697309	-	[20]
Massarinaceae	*Helminthosporium velutinum*	MFLUCC 16-1300	MG098782	MG098790	-	-	[24]
Massarinaceae	*Massarina albocarnis*	CBS119345	LC194503	LC194379	LC194337	LC194416	[66]
Massarinaceae	*Massarina cisti*	CBS 266.62 *	LC014568	AB807539	AB797249	AB808514	[19]
Massarinaceae	*Massarina cisti*	CBS 266.62	-	FJ795447	FJ795490	-	[67]
Massarinaceae	*Massarina eburnea*	CBS 473.64	AF383959	GU301840	AF164367	-	[60,68]
Massarinaceae	*Massarina eburnea*	JCM 14422	LC014569	AB521735	AB521718	AB808517	[19]
Massarinaceae	*Massarina igniaria*	CBS 845.96	-	FJ795452	FJ795494	-	[67]
Massarinaceae	*Massarina pandanicola*	MFLUCC 17-0596	MG646958	MG646947	MG646979	MG646986	[4]
Massarinaceae	*Massarina phragmiticola*	CBS 110,446	-	DQ813510	DQ813512	-	[69]
Massarinaceae	*Neottiosporina paspali*	CBS 331.37	-	EU754172	EU754073	-	[70]
Massarinaceae	*Pseudodidymosphaeria spartii*	CBS 183.58	-	GU205225	GU205250	-	[71]
Massarinaceae	*Pseudodidymosphaeria spartii*	MFLUCC 13-0273	KP325434	KP325436	KP325438	-	[72]
Massarinaceae	*Pseudodidymosphaeria spartii*	MFLUCC 14-1212	KP325435	KP325437	KP325439	-	[72]
Massarinaceae	*Pseudosplanchnonema phorcioides*	MFLUCC 14-0618	KP683372	KP683373	KP683374	-	[72]
Massarinaceae	*Pseudosplanchnonema phorcioides*	MFLUCC 13-0533	-	KM875454	KM875455	-	[73]
Massarinaceae	*Pseudosplanchnonema phorcioides*	L16	KY984360	-	KY984434	KY984467	[21]
Massarinaceae	*Pseudosplanchnonema phorcioides*	MFLUCC 13-0611	KP683375	KP683376	KP683377	-	[21]
Massarinaceae	*Semifissispora natalis*	CPC 25383	KT950846	KT950858	-	KT950878	[21]
Massarinaceae	*Semifissispora natalis*	CBS 140659	-	MH878157	-	-	[21]
Massarinaceae	*Semifissispora rotundata*	CPC 549	KT950847	KT950859	-	-	[21]
Massarinaceae	*Semifissispora tooloomensis*	CBS143431	MG38607	MG386124	-	-	[21]
Massarinaceae	*Stagonospora perfecta*	KT 1726A	AB809642	AB807579	AB797289	AB808555	[19]
Massarinaceae	*Stagonospora* cf. *paludosa*	CBS 130,005	KF251254	KF251757	-	-	[62]
Massarinaceae	*Stagonospora duoseptata*	CBS 135,093	KF251255	KF251758	-	-	[62]
Massarinaceae	*Stagonospora imperaticola*	MFLUCC 15-0026	KY706143	KY706133	KY706138	KY706146	[74]
Massarinaceae	*Stagonospora multiseptata*	MFLUCC 15-0449	KX965735	KX954404	-	-	[74]
Massarinaceae	*Stagonospora paludosa*	CBS 135088 *	KF251257	KF251760	-	KF253207	[62]
Massarinaceae	*Stagonospora perfecta*	CBS 135,099	KF251258	KF251761	-	-	[62]
Massarinaceae	*Stagonospora perfecta*	KT 1726A	AB809642	AB807579	AB797289	AB808555	[19]
Massarinaceae	*Stagonospora pseudocaricis*	CBS 135,132	KF251259	KF251763	-	-	[62]
Massarinaceae	*Stagonospora pseudopaludosa*	CPC 22,654	KF777188	KF777239	-	-	[62]
Massarinaceae	*Stagonospora pseudoperfecta*	KT 889 *	AB809641	AB807577	AB797287	AB808553	[19]
Massarinaceae	*Stagonospora* sp.	CBS 135,096	KF251263	KF251766	-	-	[62]
Massarinaceae	*Stagonospora tainanensis*	KT 1866	AB809643	AB807580	AB797290	AB808556	[19]
Massarinaceae	*Stagonospora trichophoricola*	CBS 136,764	KJ869110	KJ869168	-	-	[75]
Massarinaceae	*Stagonospora uniseptata*	CPC 22,150	KF251266	KF251769	-	-	[62]
Massarinaceae	*Stagonospora uniseptata*	CBS 135,090	KF251264	KF251767	-	-	[62]
Massarinaceae	*Suttonomyces clematidis*	MFLUCC 14-0240	-	KP842917	KP842920	-	[76]
Massarinaceae	*Suttonomyces rosae*	MFLUCC 15-0051	MG828973	MG829085	MG829185	-	[77]
Periconiaceae	*Periconia byssoides*	H 4600	LC014581	AB807570	AB797280	AB808546	[19]
Periconiaceae	*Periconia digitata*	CBS 510.77	LC014584	AB807561	AB797271	AB808537	[19]
Periconiaceae	*Periconia macrospinosa*	CBS 135,663	KP183999	KP184038	KP184080	-	[78]
Periconiaceae	*Periconia pseudodigitata*	KT 1395 *	LC014591	AB807564	AB797274	AB808540	[19]

* = The asterisks after the strain number represent the ex-type strains from the holotype specimens.

## 3. Results and Discussion

### 3.1. Phylogenetic Analyses

The individual datasets for ITS and LSU regions comprised selected isolates from closely related families (Figure 1). The RAxML analyses of the ITS dataset yielded the best-scoring trees with a final ML optimization likelihood value of -9830.778478 (Figure 1A). The matrix had 531 distinct alignment patterns with 51.80% undetermined characters or gaps. Estimated base frequencies were as follows: A = 0.227770, C = 0.273565, G = 0.243931, T = 0.254733; substitution rates AC = 2.172295, AG = 3.427213, AT = 2.029849, CG = 0.957843, CT = 5.859679, GT = 1.000000; and gamma distribution shape parameter α = 0.350193. In Figure 1A, the novel taxon *Haplohelminthosporium calami* grouped within Massarinaceae and was well separated from other genera but without good bootstrap support. *Helminthosporiella stilbacea* (MFLUCC 15-0813) is closely related to *Hel.stilbacea* (strains CPHmZC-01 and COAD 2126) with 100% ML/1.00 BYPP.

The RAxML analyses of the LSU dataset yielded the best-scoring trees with a final ML optimization likelihood value of −4283.882978 (Figure 1B). The matrix had 307 distinct alignment patterns with 12.16% undetermined characters or gaps. Estimated base frequencies were as follows: A = 0.246483, C = 0.214075, G = 0.309890, T = 0.229553; substitution rates AC = 1.828869, AG = 4.019496, AT = 3.119987, CG = 0.662100, CT = 12.098644, GT = 1.000000; and gamma distribution shape parameter α = 0.159335. In Figure 1B, the novel taxon *Haplohelminthosporium calami* was also well separated within Massarinaceae and clustered with *Helminthosporium* and *Helminthosporiella*. *Helminthosporiella stilbacea* (MFLUCC 15-0813) is closely related to *Hel. stilbacea* (strain CPHmZC-01) with 100% ML/1.00 BYPP.

The RAxML analysis of the combined (ITS, LSU, SSU, and *tef1-α*) dataset yielded a best scoring tree with a final ML optimization likelihood value of -22122.846454 (Figure 2). The matrix had 1363 distinct alignment patterns, with 41.38% undetermined characters or gaps. Estimated base frequencies were as follows: A = 0.241467, C = 0.241603, G = 0.271551, T = 0.245380; substitution rates AC = 1.860804, AG = 3.064520, AT = 1.916442, CG = 1.009390, CT = 7.530432, GT = 1.000000; and gamma distribution shape parameter α = 0.183588. In the phylogenetic analyses (Figure 2), twelve genera are included in the tree. The novel taxon of *Haplohelminthosporium calami* grouped within Massarinaceae without strong bootstrap support. *Haplohelminthosporium calami* is closely related to *H. endiandrae* (CBS 138902, MH878637), but this is statistically unsupported. *Helminthosporiella stilbacea* (MFLUCC 15-0813) constitutes a sister phylogenetic affiliation to *Hel. stilbacea* (strains CPHmZC-01 and COAD 2126) with 100% ML/1.00 BYPP statistical support.

The phylogenetic analyses (Figure 1 and Figure 2) showed several topologies of the tree had generally rather low support (ML ≤50% and BYPP ≤0.90). This reflects the relatively high amount of homoplasy in the data. Most *Helminthosporium*-like taxa did not have SSU and *tef1-α* sequence data for the phylogenetic analyses. In the future, divergent time estimations will be needed for *Helminthosporium*-like taxa to resolve taxonomic confusion and placement.

### 3.2. Taxonomy

#### 3.2.1. *Haplohelminthosporium* Konta & K.D. Hyde, gen. nov

Index Fungorum number: IF557873; Facesoffungi number: FoF09169

Etymology—Haplo in Greek means single, which refers to the single conidium in each conidiophore. It is a close relative of *Helminthosporium*.

*Saprobic* on living leaves and petioles of *Calamus* sp. On living leaves, small spots, circular to irregular, yellow in the beginning, later becoming red brown surrounded by yellow. *Colonies* on natural substrate forming black patches on the upper leaf, petiole surfaces. Sexual morph: Undetermined. Asexual morph: Hyphomycetous. *Colonies* on natural substrate forming black patches on the upper leaf, petiole surfaces. *Mycelium* mostly immersed, partly on the surface forming small stroma-like aggregations of red brown pseudoparenchymatous cells. *Conidiophores* arising singly or fasciculate from stroma cells, erect, simple, unbranched, straight, curved and swollen at apex, septate, thick-walled, cylindrical, smooth, bulbous at base, hyaline in the middle, brown to yellow-brown at 1–2-cells above the base, pale brown to yellow-brown at apical cell. *Conidiogenous cells* monotretic, terminal, determinate, cylindrical, wide and yellow-brown with a well-defined, small, noncicatrized pore at the apex. *Conidia* one for each conidiophore, obpyriform to lageniform, straight or curved, smooth, olive-brown, distoseptate, with a dark scar at the base.

Type species—*Haplohelminthosporium calami* Konta & K.D. Hyde

**Figure 3:**
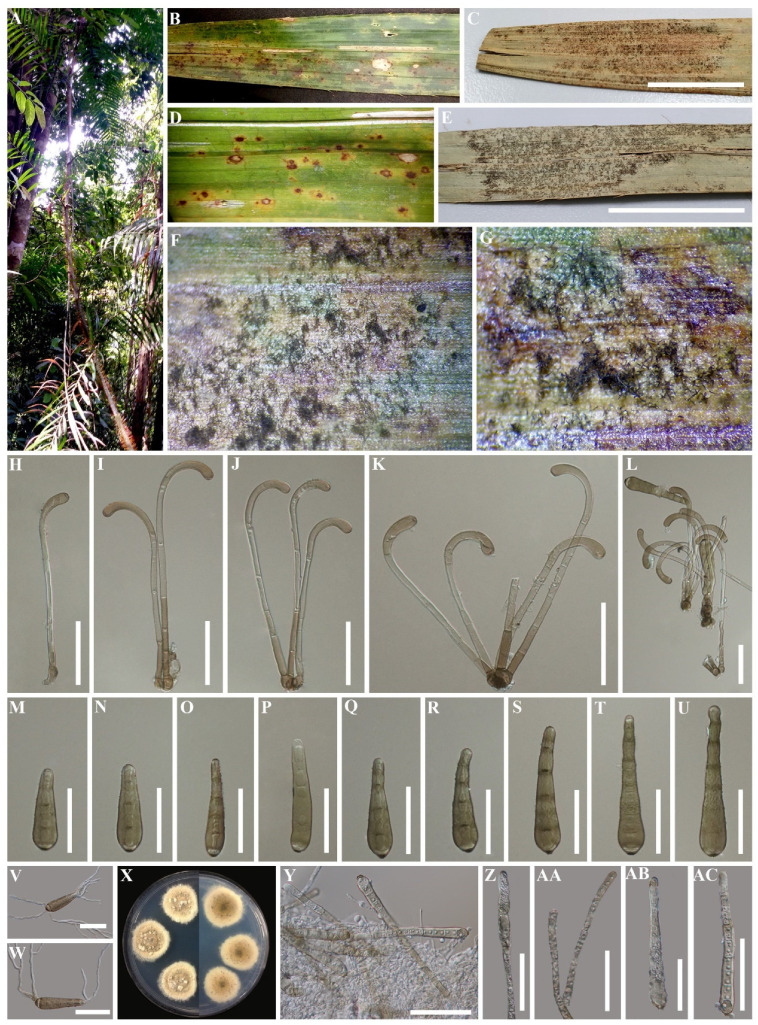
*Haplohelminthosporium calami* (MFLU 20-0520, holotype) (**A**) The forest in Krabi Province. (**B**–**E**) Fresh and herbarium palm samples. (**F,G**) Colonies on living leaf. (**H**–**L**) Conidiophores. (**M**–**U**) Conidia. (**V,W**) Germinated conidia. (**X**) Culture on PDA. (**Y**) Conidiophore and conidia on culture. (**Z**) Conidiogenesis. (**AA**) Conidiophores. (**AB,AC**) Conidia. Scale bars: C, E =2 cm, H–W, Y–AC = 50 μm.

Notes: *Haplohelminthosporium* is established as a monotypic genus with *Hap. calami* as the type species. ITS phylogenetic analyses separated this genus from other genera, while in the LSU and multigene analyses it clustered with *Helminthosporium* and *Helminthosporiella*, but both without good statistical support (Figure 1 and Figure 2). *Haplohelminthosporium* is presented herein as an asexual morph (hyphomycete) similar to *Helminthosporium* and *Helminthosporiella* in that it is hyphomycete with an erect conidiophore, monotretic conidiogenous cell and distoseptate conidia [19,22,63]. The type species of *Helminthosporium* has pale to dark brown, septate conidiophores, with terminal and intercalary polytretic conidiogenous cells, noncicatrized pores at the apex and upper 3–4 cells, solitary or short catenate conidia that are subhyaline to brown, distoseptate, and is dark brown to black scar at the base [19]. *Helminthosporiella* has brown to red-brown conidiophores with terminal, polytretic conidiogenous cells, with catenate and easily disarticulating chains of conidia that are medium brown, striated at surface and distoseptate [63]. However, *Haplohelminthosporium* is distinguished by its unbranched conidiophores arising solitarily or fasciculate from the stroma-like bulbous basal cells that are hyaline in the middle, brown to red-brown at 1–2-cells above the base, pale brown to red-brown and curved at the apical cell with well-defined non-cicatrized small pores and with a single olive-brown conidium arising from each conidiophore (Figure 3). In the BLAST search of GenBank, the closest match of the LSU, ITS, and SSU sequence data were identical to *Helminthosporium* spp. Based on distinguishing morphological characteristics together with single/multigene phylogenetic analyses we introduce the newly described strain as a new genus *Haplohelminthosporium* in Massarinaceae.

##### *Haplohelminthosporium calami* Konta & K.D. Hyde, sp. nov.

Index Fungorum number: IF557874, Facesoffungi number: FoF09170, Figure 3

Etymology: Referring to the genus of palm trees *Calamus* L.

Holotype: MFLU 20-0520.

*Saprobic* on living leaves and petioles of *Calamus* sp. On living leaves, small spots, circular to irregular, yellow in the beginning, later becoming red-brown surrounded by yellow. *Colonies* on natural substrate forming black patches on the upper leaf, petiole surfaces. Sexual morph: Undetermined. Asexual morph: *Mycelium* mostly immersed, on the surface forming small stroma-like aggregations of red brown pseudoparenchymatous stromal cells (7–)10–14(–20) μm ($\bar{x}$ = 12 μm). *Conidiophores* (110–)140–175(–215) × (4–)5–7(–8) μm ($\bar{x}$ = 160 × 6 μm, n = 50), wide at the base and apex, macronematous, mononematous, arising singly or fasciculate from the stroma cells, erect, simple, unbranched, straight, curved and swollen at the apex, thick-walled, cylindrical, smooth, bulbous at base, hyaline in the middle, brown to red-brown at 1–2-cells above the base, pale brown to red brown at the last cell of the apex, (3–)4–5(–6) septa. *Conidiogenous cells* monotretic, terminal, determinate, cylindrical, with well-defined small noncicatrized pores at the apex, wide and yellow-brown at the apex. *Conidia* (55–)70–100(–120) × (13–)17–20(–23) μm ($\bar{x}$ = 80 × 20 μm, n = 60), one on each conidiophore, obpyriform to lageniform, straight or curved, smooth, olive-brown, (3–)4–6(–7)-distoseptate, with a dark scar at the base.

Culture characteristics: Culture on PDA, colony yellow-gray-brown at the center, turning dull creamy white toward to margin, smooth, dense, zonate at the margin (Figure 3X).

Material examined: THAILAND, Krabi Province, on living leaves and petioles of *Calamus* sp. (Arecaceae), 14 December 2015, Sirinapa Konta, KHNPR-2 (MFLU 20-0520, holotype); ex-type living culture, MFLUCC 18-0074.

Notes: BLAST search of the ITS sequence of the newly described strain (*Haplohelminthosporium calami*) shows 88.89% similarity with *Helminthosporium juglandinum* (L118), the LSU sequence shows 98.75% similarity with *H. aquaticum* (MFLUCC 15-0357), and the SSU sequence shows 99.52% similarity with *H. quercinum* (L90). Based on ITS phylogenetic analysis, *Haplohelminthosporium calami* formed a single branch at the basal clades of *Helminthosporiella* and *Helminthosporium* (Figure 1A), while based on LSU analysis, *Hap. calami* clustered together with *H. juglandinum* (L97), *H. endiandrae* (CBS 138902, MH878637), and *Hel. stilbacea* with no strong statistical support for both analyses. The phylogenetic results of the combined dataset indicated that *Hap. calami* clustered with *H. endiandrae* (CBS 138902, MH878637) without strong bootstrap support (Figure 2). Comparison of base pair differences between LSU loci for isolates of *Hap. calami* strains MFLUCC 18-0074 and *H. endiandrae* strains CBS 138,902 (KP004478; Ex-type from the holotype, and MH878637; sister strain) including gaps showed 1.74% (15/861 bp) differences, and the position of each base pair difference is shown in Table 3. Other *H. endiandrae* strains (AKMR1, CBS 138902; ex-type from the holotype, and SM61) grouped together in *Helminthosporium*, as the other strains have an ITS region, but the *H. endiandrae* (CBS 138902, MH878637) strain that grouped with our new collection lacks the ITS region. Therefore, we compared the morphology of these two species and found that *Hap. calami* differs from *H. endiandrae* with respect to its smaller conidiophores ((110–)140–175(–215) × (4–)5–7(–8) vs. 200–300 × 5–7 μm), number of conidiophore septa ((3–)4–5(–6) vs. 8–16 septa), larger conidia ((55–)70–100(–120) × (13–)17–20(–23) vs. (35–)37–45(–57) × (7–)8(–9) μm), solitary conidium per conidiophore, and higher number of distoseptate ((3–)4–6(–7)-distoseptate vs. 3(–4)-distoseptate). The results show the placement of *Haplohelminthosporium calami* within Massarinaceae, and that this species is distinct from other known species. Therefore, we introduce *Hap. Calami* as a new species based on both morphological and phylogenetic data.

#### 3.2.2. *Helminthosporiella* Konta & K.D. Hyde, gen. nov.

Index Fungorum number: IF558311, Facesoffungi number: FoF09171

*Helminthosporiella* Hern.-Restr., Sarria & Crous, in Crous et al., Persoonia 36: 437 (2016), MycoBank MB816988, Nom. inval., Art. 40.3 (Shenzhen)

*Saprobic* on dead petiole of *Cocos nucifera*.Sexual morph: Undetermined. Asexualmorph:*Colony* on natural substrate black, hairy. *Mycelium* mostly immersed, at the surface forming small stroma-like aggregations of dark brown pseudoparenchymatous cells. *Conidiophores* macronematous, wide at the apex and base, arising singly from the stroma cells, erect, simple, unbranched, straight or flexuous, thick-walled, cylindrical, smooth-walled, dark brown, becoming pale brown at the apex, septate. *Conidiogenous cells* terminal and intercalary, polytretic, with well-defined thick, pale brown pores. *Conidia* obpyriform to lageniform, straight or curved, smooth-walled, subhyaline to light brown, distoseptate, with a thick scar at the base.

Type species—*Helminthosporiella stilbacea* Konta & K.D. Hyde

Notes: *Helminthosporiella* was introduced by Crous et al. [63] to accommodate a new combination of *Hel. stilbacea* Hern.-Restr., Sarria & Crous, in Massarinaceae, the basionym of the type species was not provided a Latin diagnosis [63]. In this paper we accept *Helminthosporiella* as a distinct genus, presently with a single species *Helminthosporiella stilbacea*. Since a Latin diagnosis is no longer required, we provide an English diagnosis and priority was given to the previous genus and species names. Furthermore, this study provides the holotype to validate the genus and species, and reports the first host record of *Hel. stilbacea* associated with coconut tree (Arecaceae) in Thailand. In particular, based on the present morphology and DNA sequence data, *Helminthosporiella* is identified as a monotypic genus, with *Hel. stilbacea* as the type species. The members of *Helminthosporiella* were found associated with leaf spots on oil palm (Arecaceae) [64].

##### *Helminthosporiella stilbacea* Konta & K.D. Hyde, sp. nov.

**Figure 4:**
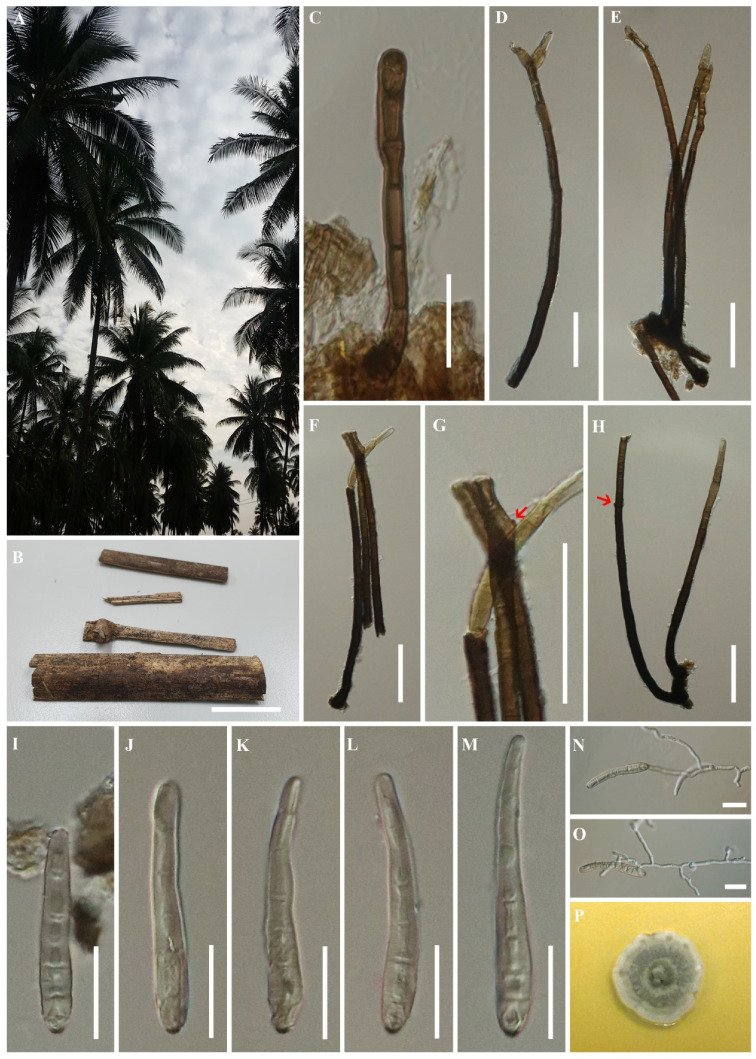
*Helminthosporiella stilbacea* (MFLU 20-0521, holotype) (**A**) A coconut plantation in Prachuap Khiri Khan Province. (**B**) Palm samples. (**C**–**E**) Conidiogenesis. (**F**–**H**) Conidiophores (at red arrow are pores). (**I**–**M**) Conidia. (**N,O**) Germinated conidia. (**P**) Culture on MEA. Scale bars: B = 2 cm, C, I–O = 20 μm, D–H = 50 μm.

Index Fungorum number: IF558312, Facesoffungi number: FoF09172, Figure 4.

=*Cercospora palmicola* f. *stilbacea* Moreau, Rev. Mycol. 12: 38. 1947 Nom. inval., Art. 39.1 (Shenzhen)

≡*Helminthosporiella stilbacea* Hern.-Restr., Sarria & Crous, in Crous et al., Persoonia 36: 437. 2016; Nom. inval., Art. 39.1 (Shenzhen)

*Helminthosporium stilbaceum* Moreau ex S. Hughes, Mycol. Pap.48: 38. 1952; Nom. inval., Art. 39.1 (Shenzhen).

≡*Exosporium stilbaceum* Moreau ex M.B. Ellis, Mycol. Pap.82: 38. 1961; Nom. inval., Art. 39.1 (Shenzhen).

=*Exosporium stilbaceum* var. *macrosporum* Subramon. & V.G. Rao, Journal of the Annamalai University, part B, Sciences 29: 404. 1971; Nom. inval., Art. 35.1 (Shenzhen).

*Saprobic* on dead petiole of *Cocos nucifera*.Sexual morph: Undetermined. Asexualmorph: *Colony* on natural substrate black, hairy. *Mycelium* mostly immersed, at the surface forming small stroma-like aggregations of dark brown pseudoparenchymatous cells (6–)11–15(–25) μm diam ($\bar{x}$ = 14 μm). *Conidiophores* (60–)165–270(–310) × (5–)7–9(–12) μm ($\bar{x}$ = 200 × 8 μm, n = 30), macronematous, wide at the apex and base, arising singly from the stroma cells, erect, simple, unbranched, straight or flexuous, thick-walled, cylindrical, smooth-walled, dark brown, becoming pale brown at the apex, (4–)12–15-septate. *Conidiogenous cells* terminal and intercalary, polytretic, with well-defined thick, pale brown pores. *Conidia* (30–)45–60(–70) × 6–9 μm ($\bar{x}$ = 50 × 7 μm, n = 30), obpyriform to lageniform, straight or curved, smooth-walled, subhyaline to light brown, 5–8-distoseptate, with a thick scar at the base.

Culture characteristics: Culture on MEA, colony yellow-green at the center, turning dull green, pale yellow next, becoming dull green again, pale yellow, and white toward the margin. Colony smooth, dense at the middle, zonate, fluffy at the margin (Figure 4P).

Material examined: THAILAND, Prachuap Khiri Khan Province, on dead petiole of *Cocos nucifera* L. (Arecaceae), 30 July 2015, Sirinapa Konta PJK04gHB (MFLU 20-0521, holotype); ex-type living culture, MFLUCC 15-0813.

Notes: Crous et al. [63] introduced a new genus *Helminthosporiella* with a new combination of *Hel. stilbacea* based on fresh collections from oil palm (*Elaeis oleifera*) in Colombia and the second collection of *Hel. stilbacea* was also collected from oil palm (*Elaeis guineensis*) in Brazil by Rosado et al. [64]. The full descriptions, illustrations, and sequence data are provided with interesting information as this species causes elliptical necrotic spots with a yellowish halo on living leaves of commercial oil palm plantations [63,64]. However, the type species was invalid because of the basionym lacked a Latin diagnosis [63]. From these, our fresh collection was collected from dead petiole of coconut (*Cocos nucifera*) and in phylogenetic analysis (Figure 1 and Figure 2), three strains of *Hel. stilbacea*, including our strain, are grouped together with high bootstrap support. In this study, we therefore provide a holotype from our specimen, and introduce a new species *Helminthosporiella stilbacea*, complete with an English diagnosis, and validated by using the same name while linking to the valuable information provided from the previous publication of this species.

A BLAST search of the ITS sequence of our isolate showed 90.19% similarity with *H. velutinum* (L131), the LSU sequence showed 97.05% similarity with *H. aquaticum* (MFLUCC 15-0357), the SSU sequence showed 99.15% similarity with *H. quercinum* (L90), and the *tef1-α* sequence showed 92.61% similarity with *H. tiliae* (L88). These blast results do not match the results of the phylogenetic analyses.

The comparison between three strains of *Hel.stilbacea* (see Table 4) from three collections showed that our collection MFLU 20-0521 has several differences when compared with the other two strains CPHmZC-01 and COAD 2126. Our collection was obtained from a dead petiole, while the two other strains were isolated from living leaves [63,64]. Therefore, our new collection has been provided as a holotype for *Hel. Stilbacea*. It is also the first geographical record from Thailand, and is a new record of the species from a coconut host (*Cocos nucifera*).

## 4. Conclusions

In this study, we introduce the new genus *Haplohelminthosporium*,with *Hap. calami* as the type species. In multigene phylogenetic analyses, *Hap.calami* clustered together with *Helminthosporium endiandrae* (CBS 138902) without strong good bootstrap support (other *H. endiandrae* (AKRM1, CBS 138902 (ex-type), SM61) groups together in *Helminthosporium*). Moreover, we were unable to synonymize *H. endiandrae* (CBS 138902) under *Haplohelminthosporium* because *H. endiandrae* has only LSU sequence data available [60]. In the future, *H. endiandrae* needs more collections and sequence data to confirm taxonomic placement.

Another newly described isolate clusters together with *Helminthosporiella stilbacea. Helminthosporiella* was introduced by Crous et al. [63] but was invalidated as the type species was not provided with a Latin diagnosis. In this study, we validate *Helminthosporiella* with *Hel. stilbacea* as the type species. Moreover, the newly described strain from this study is the first saprobic report of *Hel. stilbacea*, as this was reported in previous studies as a pathogenic fungus on leaves [63,64]. Moreover, topological nodes in phylogenic analyses showed conflicting results (Figure 1 and Figure 2). Probably, using only single gene ITS or LSU analyses will preclude the establishment of taxonomic placements, while combined gene analyses (including protein coding genes) provide sufficient molecular data to determine the placements.

*Helminthosporium* is generally described as a common saprobe found on leaf or twig litter, and it appears to have a diverse distribution. Occasionally, members of this genus are also described as pathogens, occurring on a wide range of hosts. Comparison of morphology is important for fungal identification [79]. In this study, we provide a checklist for *Helminthosporium* species reported worldwide including details of each species based on records from Species Fungorum [80] (Table 5). We noted that ten *Helminthosporium* species have been found on palm substrates (Arecaceae). Although *Helminthosporium* conidia superficially resemble many genera, such as *Drechslera*, *Bipolaris*, and *Exserohilum*, phylogenetic analyses have provided different results [19,33,81,82,83]. Furthermore, we recommend revision of the genus *Helminthosporium* with fresh collections and DNA sequence data (specifically the ITS region and protein coding genes).

## Figures and Tables

**Table 1:** Details of genes/loci with PCR primers and PCR conditions.

Genes/loci	PCR Primer (Forward/Reverse)	PCR Conditions
LSU	LR0R/LR5	^a^; 95 °C: 30 s, 55 °C: 50 s, 72 °C: 30 s (35 cycles); ^b^
ITS	ITS5/ITS4	
SSU	NS1/NS4	
*tef1-α*	983F/2218R	

^a^ Initiation step of 95 °C: 3 min; ^b^ Final elongation step of 72 °C: 10 min and final hold at 4 °C.

**Table 3:** Polymorphic nucleotides from sequence data of the LSU loci for isolates of *Haplohelminthosporium calami* MFLUCC 18-0074 and *Helminthosporium endiandrae* CBS 138,902 (KP004478, MH878637).

Species	Strain	LSU														
		6	34	74	270	400	412	419	427	480	484	490	491	524	644	843
*Haplohelminthosporium calami* (this study)	MFLUCC 18-0074	-	A	A	T	T	T	C	C	A	C	A	T	T	T	G
*Helminthosporium endiandrae* (Ex-type from the holotype)	CBS 138,902 (KP004478)	-	C	C	C	C	C	T	T	C	T	T	G	C	G	G
*H. endiandrae* (sister strain in Figure 1B and Figure 2)	CBS 138,902 (MH878637)	C	A	C	C	C	C	T	T	C	T	T	G	C	G	-

**Table 4:** Comparison of three strains of *Helminthosporiella stilbacea*.

No.	Herbarium/ Culture No.	Host (Genus/Family)	Locality	Morphology				References
				Mycelia (μmWide)	Conidiophores (μm)	Conidiogenous Cells (μm)	Conidia (μm)	
1.	Herbarium: - Culture no.: CPHmZC-01	On leaves of *Elaeis oleifera*/Arecaceae	Colombia	Hyaline to pale brown, smooth, branched, septate	Erect, brown to red-brown, synnematous, septate, compacted, 620–1400 × 19–54, individual hyphae 3–4 wide	Mono- or polytretic, integrated, determinate, terminal, cylindrical, 31–67 × 4.5–7	Catenate, obclavate, subcylindrical, occasionally bifurcate, medium brown, 26–83 × 7–10, (1–)3–5(–6)-distoseptate	[63]
2.	Herbarium: - Culture no.: COAD 2126	On old leaves of *Elaeis* guineensis/Arecaceae	Brazil	Hyaline to pale brown, 2–4	Erect, brown, septate, synnematous, 66–201(−770) × 2.5–6(−18)	Mono or polytretic, cylindrical, terminal, 18–59 × 4–7	Catenate, subcylindrical, obclavate, brown, 32–83 × 4–11, 2–7-distoseptate	[64]
3.	Herbarium: MFLU 20-0521 Culture no.: MFLUCC 15-0813	On dead petiole of *Cocos nucifera*/Arecaceae	Thailand	Mostly immersed, dark brown	Solitarily, erect, unbranched, straight or flexuous, cylindrical, bulbous at base, dark brown, becoming pale brown at the apex, (60–)165–270(–310), (5–)7–9(–12) at the base, 5–8 μm wide at the apex, (4–)12–15 septate	Terminal and intercalary with well-defined pores, pale brown	Obpyriform to lageniform, straight or curved, light brown, (30–)45–60(–70) × 6–9, 5–8-distoseptate	This study

**Table 5:** Morphology, host information, locality, sequence data, and related references of *Helminthosporium* reported worldwide based on the record of Species Fungorum 2021 (bold text present *Helminthosporium* reported from Arecaceae).

No.	Taxa	Host(Genus/Family)	Locality	Morphology	Sequence Data	References
1	*H. abietis*	*Abies* sp./Pinaceae	U.S.A./Washington	*Conidiophores* irregularly branched; *Conidia* 126–150 × 12–16 µm, fusiform, pointed at both ends, olive-green, 12–15-distoseptate	Absent	[84]
2	*H. acaciae*	On dead branches of *Acacia farnesiana*/ Fabaceae	Sierra Leone	*Conidiophores* 140–280 × 7–11 µm, dense, fasiculate, simple, straight or flexuous, sometimes swollen at at the tip, septate, smooth, thick-walled, brown, with well-difinded small pores at the apex; *Conidia* 31–(44–)49 × 10–(12–)14 µm in widest part, narrowing towards the apex to 3–5 µm, obclavate, straight or flexuous, smooth-walled subhyaline to pale brown, 3–6-distoseptate, with a small dark blackish-brown to black scar at the base	Absent	[85]
3	*H. acalyphae*	On leaves of *Acalypha angustifolia*/ Euphorbiaceae	Dominican Republic	*Conidiophores* 2.5–4 µm thick, erect, simple, superficial, brown-blackish, septate; *Conidia* 9–16 × 4–6 µm, one for each conidiophore, ovate-ellipsoid, olivaceous-brown or dull-brown, 2–3-distoseptate	Absent	[86]
4	*H. accedens*	On living leaves of *Dolichos baumii*/Fabaceae	Namibia	*Conidiophores* 250–300 × 5–9 µm, erect, olive-brown; *Conidia* 35–57 × 6.5–9 µm, solitary, oblong-fusoid, olive, 3–6-distoseptate	Absent	[87]
5	*H. ahmadii*	On dead branches of *Quercus* sp./Fagaceae	Pakistan	*Conidiophores* 220–650 × 12–15 µm, dense, fasiculate, simple, straight or flexuous, smooth, thick-walled, brown to dark brown, with small pores at the apex, septate; *Conidia* 95–(110–)150 × 25–30(–38) µm wide inthe broadest part, tapering towards the apex to 5–9 µm, obclavate, sometimes rostrate, straight or flexuous, smooth-walled, brown or dark brown, 5–15-distoseptate, with a dark blackish-brown to black scar at base	Absent	[85]
6	*H. aichrysonis*	On leaves of *Aichryson dichotomum*/ Crassulaceae	Spain	No information available	Absent	[88]
7	*H. alatum*	On dying leaves of *Dioscorea alata*/ Dioscoreaceae	Dominican Republic	No information available	Absent	[89]
8	*H. albiziae*	On leaves of *Albizia lebbeck*/Fabaceae	Sri Lanka	*Conidiophores* 70 ×7 µm; *Conidia* 42–56 × 12 µm, tapering to 4 µm diam. clavate, ends rounded, at the lower end, rough with minute warts, fuliginous, terminal cell paler, strgight or curved below, 3–4-distoseptate	Absent	[90]
9	*H. albiziicola*	*Albizzia lebbek*/Fabaceae	India	*Conidiophores* 28–44 × 4.5–6 µm, straight or slightly curved, one-septate at the base; *Conidia* 23.5–34 × 8–9 µm, pyriform, prolongate at the apex, rounded at the base, pale, cinnamon-brown, 3-distoseptate	Absent	[91]
10	*H. allamandae*	On living leaves of *Allamanda cathartica*/ Apocynaceae	Dominican Republic	*Conidiophores* 100–180 × 8–10 µm, solitary or aggregate, curved, simple, dark-brown; *Conidia* 66–110 × 17–20 µm, clavate, elongate-ellipsoid or subfusoid, erect or curved, gray-brown, 7–10-distoseptate	Absent	[92]
11	*H. alphitoniae*	On living leaves of *Alphitonia* sp./ Rhamnaceae	Malaysia/Mount Kinabalu	*Conidiophores* 250–500 ×5–8 µm, erect, dark-brown; *Conidia* 25–66 × 8–13 µm, obclavate, erect or curved, yellow-brown or pale olive, 1–6-distoseptate	Absent	[93]
12	*H. aneurolepidii*	On leaves of *Aneurolepidium ramosum*/Poaceae	Russia/West Siberia	No information available	Absent	[94]
13	*H. anomalum*	From soil	U.S.A./Iowa, Utah	No information available	Present	[17,63]
14	*H. anonymicum*	In culture: former Soviet Union	Russia	No information available	Absent	[95]
15	*H. apiculatum*	On dry tree of *Betula* sp. (*Betulinum*)/ Betulaceae	Czech Republic	*Conidiophores* fasiculate, flexuous, simple, hyaline; *Conidia* long, 37 μm, elliptical-fusiform, with color, multi-septate	Absent	[96]
16	*H. appatternae*	From leaves of *Cynodon dactylon*/Poaceae; from culture	India/Maharashtra	*Conidiophores* unbranched, of two types; determinate conidiophores uniform, 182 × 5.2 μm, single, olivaceous, 1–3 septate; indeterminante conidiophores narrower, 208–520 × 7.8 μm, paler and distantly septate at base, gradually broadened into a darker, close septate; *Conidia* 20.8–152.0 × 7.8 μm, 6–18-distoseptate	Absent	[18]
17	*H. appendiculatum*	On branches of the trees	Czechia	*Conidiophores* simple, fasciculate; *Conidia* 65 × 11 μm, clavate, curved, blunted, whitish, multi-septate	Absent	[96]
18	*H. aquaticum*	On submerged decaying wood	China/Yunnan	*Conidiophores* 410–580 × 13–17 μm, solitary or in groups of 2–4, erect, flexuous, unbranched, smooth, dark brown paler towards the apex, bulbous at base, 14–23 septate; *Conidia* 70–80 × 16–18 μm, single, obclavate, straight or curved, pale brown to brown, truncate and cicatrized at base, wider than apex, guttulate, 8–10-distoseptate	Present	[20]
19	*H. arcautei*	On living leaves *Scorpiurus subvillosa*/ Fabaceae	Spain	*Conidiophores* 35–50 × 7–8 μm, erect, simple, cylindrical, brownish-purple, 2–3 septate; *Conidia* 48–86 × 10.5–11 μm, cylindrical-fusoid, straight or slightly curved, light-brown chestnut, 3–8-distoseptate	Absent	[95,97]
20	*H. asterinoides*	On living leaves of *Eugenia* sp./Myrtaceae	Brazil	*Conidiophores* 5–7 μm thick, fasciculate, rhizoid; *Conidia* 22–24 × 5–6 μm, fusoid, curved, colorless at each bottom, 3-distoseptate	Absent	[98]
21	*H. asterinum*	On *Liquidambar* sp./ Altingiaceae	U.S.A./Florida	*Conidiophores* erect, simple, septate; *Conidia* 500–600 × 80 μm, clavate, 3–4-distoseptate	Present	[99]
22	*H. astragali*	On leaves of *Astragalus siversianus*/ Fabaceae	Kyrgyzstan	No information available	Absent	[100]
23	*H. atypicum*	On leaves of *Triticum* sp./Poaceae	India/Maharashtra	*Conidiophores* 3–7 septate, unbranched, and of two types; shorter conidiophore uniformly wide, 62.4–72.8 × 7.8 μm, brown; longer ones narrow at the base and paler, gradually broadening and darkening towards the apex, 440–680 × 5.2–10 μm; *Conidia* yellow to brown, darkening at maturity, of two kinds; normal ones 23–93.6 × 26 μm, elliptical with hemispherical edges, widest at the middle, 0–10-distoseptate; a typical conidia abundant, forked or geniculate, septation forked, brown to dark brown, 5–8-distoseptate	Absent	[101]
24	*H. austriacum*	On dead corticated twigs of *Fagus sylvatica*/Fagaceae	Austria/Döbling, Kahlenberg, Wien	*Conidiophores* 275–700(–920) μm long, 11.5–19 μm wide at the base, tapering to 7–11 μm near the apex, solitarily or fasciculate, erect, simple, sub-cylindrical, straight or flexuous, thick-walled, smooth, brown to dark brown, paler near the apex, with well-defined small pores at the apex, 1–12 septate; *Conidia* (30–)35–48(–97) × (10.0–)13.7–16.5(–19.8) μm, tapering to 4.5–6.0 μm at the distal end, obpyriform to lageniform, straight or curved, smooth, pale brown, (4–)5–7(–10)-distoseptate, with a blackish-brown 3–6 μm wide scar at the base	Present	[21]
25	*H. avenae-pratensis*	On sheaths of *Avena pratensis*/Poaceae	Germany	*Conidiophores* 300 × 8–11 μm, solitary or fasciculate, dark-chestnut, septate; *Conidia* 70–107 × 16–21 μm, cylindrical or obclavate, light brown, on both sides paler, 5–11-distoseptate	Absent	[102]
26	*H. bactridis*	On sheaths of *Bactris* sp./Arecaceae	Brazil/Pará	*Conidiophores* 200 × 3–4.5 μm, septate; *Conidia* 20–30 × 6–8 μm, fusoid, 6–7-distoseptate	Absent	[103]
27	*H. bakeri*	On dead stems of *Premnavestita* sp./ Lamiaceae	Philippines	*Conidiophores* 500–800 ×12 μm wide at base to below, 10 μm wide, erect, unbranched, dark; *Conidia* 80–150 × 17–22 μm, solitary, oblong, obclavate, 3–6-distoseptate	Absent	[104]
28	*H. bambusicola*	On dead culm of *Bambusa* sp./Poaceae	China/Sichuan	*Conidiophores* 55–247 × 4–6 μm, fasciculate or solitary, simple, cylindrical, straight or flexuous, thick walled, smooth, brown, paler towards the apex, with well-defined small pores, 1–2 septate; *Conidia* 36–66 × 6–11 μm narrowing towards the apex to 2–4.5 μm wide, obclavate, straight or slightly flexuous, thin-walled 1–1.5 μm thick, smooth, pale brown, paler towards the apex, 5–8-distoseptate, scar not distinct at the base	Absent	[105]
29	*H. bataticola*	On living leaves of *Ipomoea batatas*/ Convolvulaceae	Caucasus	No information available	Absent	[106]
30	*H. bauhiniae*	On dead twigs of *Bauhinia tomentosa*/ Fabaceae	Sierra Leone	*Conidiophores* 350–110 × 10–15 μm thick at the apex, 15–20 μm thick at the base, dense, fasciculate, simple, straight or flexuous, smooth-walled, dark brown, sometimes paler towards the apex, with well definded, small pores septate; *Conidia* 55-(86–)145 × 16–(17.2–)18 μm thick in broadest part, tapering to 3–4 μm the apex, obclavate, straight or flexuous, rostrate, smooth-walled, subhyaline to brown, 7–18-distoseptate, with a dark blackish brown to black scar ath the base	Absent	[85]
31	*H. belgaumense*	On litter, *Calamus thwaitesii*/Arecaceae	India/Karnataka	*Conidiophores* 140–250 × 6–9 μm, erect, straight to flexuous, unbranched, smooth, brown; *Conidia* 10–15 × 6–11 μm, solitary, dry, sub-spherical, dark brown, truncate at base, roundea at the apex, 1-distoseptate	Absent	[107]
32	*H. bhawanii*	On leaves of *Eragrostis japonica*/Poaceae	India/Bihar	No information available	Absent	[108]
33	*H. bigenum*	Palmae rotten petiole/Arecaceae	Peru	No information available	Absent	[109]
34	*H. bondarzewii*	From grains of *Triticum* sp. and *Secale* sp./ Poaceae	Russia, Ukraine	No information available	Present	[60,110]
35	*H. cacaliae*	*Cacalia sonchifolia*/Asteraceae	Brazil	No information available	Absent	[111]
36	*H. cacaophilum*	From unfermented Cacao beans, *Theobroma cacao*/Malvaceae	Dominican Republic/Santo Domingo	No information available	Absent	[112]
37	*H. cactacearum*	In young plants of *Cereus* species/Cactaceae	Italy	No information available	Absent	[113]
38	*H. caespitiferum*	Meliola spec. in leaf spots of living leafs of *Omphalea pauciflora*/ Euphorbiaceae	Dominican Republic/Santo Domingo	*Conidiophores* 150–300 × 6.5–8 μm, simple, dark-brown, septate; *Conidia* 18–42 × 8–11 μm, oblong to fusoid, dark-brown, constrict at septum, (3–)6–7-distoseptate	Absent	[92]
39	*H. canephorae*	*Coffea canephora*/Rubiaceae	Democratic Republic of the Congo/Zaire	No information available	Absent	[114]
40	*H. cantareirense*	On dead stems	Brazil/São Paulo	*Conidiophores* 7–12 μm thick, erect, fasciculate; *Conidia* 50–60 × 8–12 μm, clavate, brown, constrict at septum, 6–8-distoseptate	Absent	[115]
41	*H. cantonense*	On decaying culms of *Bambusa vulgaris*/Poaceae	China	*Conidiophores* 80–95 × 6 μm; *Conidia* 50–62 × 8 μm, obclavate, 7–9-distoseptate	Absent	[116]
42	*H. caperoniae*	On living leaves of *Caperonia palustris*/Euphorbiaceae	Dominican Republic	*Conidiophores* 100–300 × 3.5–5 μm, 2–5 fasciculate, simple, olive-brown; *Conidia* 22–55 × 4–6 μm, oblong-fusoid or subclavate, rarely cylindrical, yellow or gray-brown	Absent	[92]
43	*H. carpocrinum*	Parasite on perithecia of *Meliola funebris* on leaves of *Omphalea* sp./Euphorbiaceae (*O. pauciflora*)	Dominican Republic/Santo Domingo	*Conidiophores* 1–4 articulate, 200–350 μm long, very densely fasciculate, erect to sub-erect, straight or slightly irregularly curved, almost straight ot curved, dark-brown to blackish, tip light-colored; *Conidia* 22–25 ×8–10 μm, 1–4 to each conidiopore, easily falling, ellipsoid to ovoid, with narrowed ends, or basal end narrowed-truncate, apical end rounded to acute, not caudate, central cells from dark-brown to brownish, and cells light brown to yellowish, 2–5-distoseptate	Absent	[117]
44	*H. carposaprum*	On *Lycopersicon esculentum*/Solanaceae	British Guiana, Haiti, Mexico	No information available	Absent	[118]
45	*H. ceibae*	On leaves of *Ceiba pentandra*/Malvaceae	Philippines	No information available	Absent	[119]
46	*H. chlorophorae*	On dead twigs of *Chlorophora regia*/Moraceae	Sierra Leone	*Conidiophores* 120–270 × 7–10 μm thick at the base, often swollen towards the tip up to 12 μm, single or fasciculate, simple, straight or flexuous, smooth-walled, brown to dark brown, with 1–3 well-definded, small pores, septate; *Conidia* 52–(73–)102 × 8–(9.5–)11 μm, thick in the widest part narrowing gradually towards the apex to 3–5 μm, obclavate, straight or flexuous, smooth-walled, subhyaline to pale brown, 6–9-distoseptate, with a tather large dark blackish-brown to black scar at the base	Present	[85,120]
47	*H. chrysobalani*	On dry leaves of *Chrysobalanus icaco*/ Chrysobalanaceae	Dominican Republic/Bonao	*Conidiophores* up to 6 μm, fasciculate, erect, 2–3 septate; *Conidia* 25–50 × 3–4 μm, fusoid, 2–4-distoseptate	Absent	[121]
48	*H. chusqueae*	On living and dying leaves of *Chusquea serrulata*/Poaceae	Ecuador/Tungurahua	*Conidiophores* 200–350 × 4–6 μm, dense, erect, fasciculate, simple, straight or slightly curved, dark-brown or olive, septate; *Conidia* 32–50 × 9–11 μm, elongate-fusiform, blunt at both ends, curved, rarly straight, gray or olive-brown, 3–4-distoseptate	Absent	[122]
49	*H. cibotii*	On leaves of *Cibotium* sp./Cibotiaceae	U.S.A./Hawaii Islands	No information available	Absent	[123]
50	*H. ciliare*	-	-	No information available	Absent	[124]
51	*H. citri*	On leaves of *Citrus poonensis*, *Citrus tankart*, *Citrus ponki*, and of *Citrus sinensis* var. *brasiliensis*/Rutaceae	China/Taiwan	No information available	Absent	[125]
52	*H. claviphorum*	Rotten branch	Peru	No information available	Absent	[109]
53	*H. cleosmatis*	On living leaves of *Clematis* sp./ Ranunculaceae (in foliisvivis *Cleosmati soctandri*)	Dominican Republic	*Conidiophores* 140–250(–300) μm long, 4–5 μm wide, solitary, erect, simple, dark-brown, often becoming paler; *Conidia* 28–52 × 6.5–9 μm, clavate or fusoid, yellow or pale olive-brownish, (3–)4–5-distoseptate	Absent	[92]
54	*H. clusiae*	On leaves of *Clusiarosa* sp./Clusiaceae	Dominican Republic	*Conidiophores* 108–128 × 12–16.5 μm effuse, brown-black, irregular at based, or subbulbose, septate; *Conidia* 26–32 × 10–11.5 μm, fusoid, subfusoid or cylindrical, 4–8-distoseptate	Absent	[126]
55	*H. coffeae*	On leaves of *Coffea liberica*/Rubiaceae	Ghana	*Conidiophores* 300–400 × 7–8 μm, effuse, nigro-olivaceas, aggregate, erect, cylindrical, rect or flexuous, olives-brown, septate; *Conidia* 45–55 × 8–10 μm, obovate, 3–5-distoseptate	Absent	[127]
56	*H. conidiophorellum*	On dead branches of tree	China/Guangxi	*Conidiophores* 60–280 × 7.0–8.5 μm, fasciculate, simple, subcylindrical, straight or flexuous, thick-walled, smooth, dark brown, paler towards the apex, with 1–3 well-defined small pores at the apex, 1–2 septate; *Conidia* 100–147.5 μm long, 9.5–11 μm diam in the widest part, narrowing towards the apex to 3–4 μm diam, straight or slightly flexuous, smooth-walled, pale brown, sometimes verruculose at apex, 11–17-distoseptate, with a large dark blackish-brown scar at the base, 2–3 μm thick	Absent	[128]
57	*H. constrictum*	On dead branches of *Trachycarpus fortunei*/Arecaceae	China/Guangdong	*Conidiophores* single, simple, subcylindrical, straight or slightly flexuous, brown to dark brown, paler towards the apex, 1–3 septate; *Conidia* 57–120 × 9–12 µm, thick in the widest part, narrowing toward the apex to 2.5–5 µm, abruptly tapered to a truncate base, tretic, obclavate, straight or slightly flexuous, pale brown, paler toward to apex, 9–15-distoseptate, sometimes constricted at one or two septa	Absent	[129]
58	*H. conviva*	On *Hyphoderma caliciferum*, the genus of crust fungi in the family Meruliaceae.	Spain/Archipelago/Balearic/Baleares Islands	No information available	Absent	[130]
59	*H. corchori*	On leaves of *Corchorus capsularis*/Malvaceae	China/Taiwan	No information available	Absent	[131]
60	*H. crassiseptum*	*Meliola abrupta*	Dominican Republic	*Conidiophores* 30–50 × 2–3 µm, septate; *Conidia* 45–55(–65) × 12–14 µm, ovoid or elliptical, (2–)3-distoseptate	Absent	[86]
61	*H. crotalariae*	On leavesof *Crotalaria juncea*/Fabaceae	India/Assam	No information available	Absent	[132]
62	*H. crus-galli*	On living leaves of *Echinochloa crus-galli* (=*Panicum crista-galli*)/Poaceae	Japan	No information available	Absent	[133,134]
63	*H. cubense*	On rachis of *Roystonea regia*/Arecaceae	Cuba	No information available	Absent	[135]
64	*H. cucumerinum*	On living leaves of *Cucumis sativus*/ Zingiberaceae	Russia/Krym	No information available	Absent	[136]
65	*H. curvulum*	On decaying leaves of *Zea mays*/Poaceae	Philippines	*Conidiophores* 160–180 × 7–7.5 µm, fasciculate, filiform, septate; *Conidia* 25–35 × 8–9 µm, oblong-fusoid, narrow, 3(–4)-distoseptate	Absent	[137]
66	*H. cuspidatum*	On decaying branches of *Afzelia rhomboidea*/Fabaceae	Philippines	*Conidiophores* 800–900 × 8–9 µm, fasciculate, filiform, multiseptate; *Conidia* 100–130 × 11–12 µm, obclavate, 8–12-distoseptate	Absent	[137]
67	*H. cylindricum*	On rotten wood	Czech Republic/Bohemia	*Conidiophores* 100–130 × 4–5 µm, subfasciculate, filiform long, simple, fuliginous up paler, septate; *Conidia* 14–15 × 2.5 µm, cylindrical, apex rounded, base acuted, minute, pale fuliginous, 3-distoseptate	Absent	[138]
68	*H. cymmartinii*	On leaves of *Cymbopogon martinii*/Poaceae	India/Uttar Pradesh	No information available	Absent	[108]
69	*H. cyperi*	On *Cyperus* sp./ Cyperaceae	Greece	*Conidiophores* straight to subflexuous, greenish, paler at apex; *Conidia* 78 × 9 µm, fusoid, fuscidull, 5–8-distoseptate	Absent	[139]
70	*H. dactylidis*	On leaves of *Dactylis glomerata*/Poaceae	U.S.A./Pennsylvania	No information available	Absent	[140]
71	*H. dalbergiae*	On dead branches of *Dalbergia sissoo*/Fabaceae	Pakistan	*Conidiophores* 300–1300 × 10–12(–15) µm, dense, fasciculate, simple, flexuous, smooth-walled, brown to dark brown, sometimes paler towards the apex, with well-definded small pores, septate; *Conidia* 58–(93–)125 × 12–(13.2–)14 µm thick in broadest part, tapering to gradually towards the apex to 3–5 µm, obclavate, straight or flexuous, smooth-walled, straw-coloured to pale brownwith, 5–17-distoseptate, large dark blackish-brown to black scar at the base	Present	[85]
72	*H. davillae*	On leaves of *Davilla rugosa*/Dilleniaceae	U.S.A./San Francisco	*Conidiophores* 4–6 µm, thick filiform, flexuous, unbranched, elongate, brown, septate; *Conidia* 40–70 × 4–6 µm, elongate-obclavate, narrower and paler, (1–)2–4-distoseptate	Absent	[141]
73	*H. decacuminatum*	In the dry twigs on *Vitis vinifera*/Vitaceae	Italy	*Conidiophores* 4 µm thick, extremely short-articulated, irregular, dark reddish-brown; *Conidia* 40–45 × 10 µm, long clavate, decacumina to tip, or cut down in pedicellum narrowed, pale brown-gray, 4–5-distoseptate	Present	[60,142]
74	*H. delicatulum*	On stems of Umbelliferae or Apiaceae	UK/Great Britain	*Conidiophores* slender, subulate, multi-articultate, brown, paler at the tips; *Conidia* oblong, nearly colourless, with the apices very obtuse, consisting of about five swollen articulations, one or two of which have occasionally a vertical dissepiment	Absent	[143]
75	*H. delphinii*	On stems of *Delphinium brunonianum*/ Ranunculaceae	Russia	No information available	Absent	[144]
76	*H. dendroideum*	On *Acer* sp./ Sapindaceae	U.S.A./South Carolina	*Conidiophores* 1–2 short branchlets termintated, oblong, subfusiform, slightly curved, multiarticulate conidia; *Conidia* 60 µm long, each joint containing a globose nucleus	Absent	[145]
77	*H. densum*	-	-	No information available	Absent	[146]
78	*H. desmodii*	On *Desmodium buergeri*/ Fabaceae	Japan	No information available	Absent	[147]
79	*H. diedickei*	No information available	No information available	No information available	Absent	[148]
80	*H. dimorphosporum*	On decaying rotting stems of unknown liana	Cuba	*Conidiophores* 150–400 µm long, at the apex 9–12 µm, at the base 10–14 µm wide, single or fasciculate 2–10, simple, straight or flexuous, smooth, dark brown, paler towards the apex, septate; *Conidia* of two different types arising through pores a t the apex (1–4 pores) and late rally beneath the upper septa: (a) 19–24 × 8–10.5 µm, broadly ellipsoidal, ovoid or broadly fusiform, thick-walled, smooth, brown to dark brown, 1-distoseptate; (b) 24–65 µm long, 10–15 µm wide in the broadest part, tapering to 3.2–4.8 µm at the apex, obclavate, rostrate, straight or flexuous, pale brown, smooth, 6–9-distoseptate, with a dark brown scar at the base	Absent	[149]
81	*H. dolichi*	On living leaves of *Dolichos* sp./Fabaceae	Namibia	*Conidiophores* 250–350 × 4–6 µm, erect, olive-brown; *Conidia* 27–38 × 5.5–8 µm, solitary, oblong-subfusoid, olive, 2–3-distoseptate	Absent	[87]
82	*H. dongxingense*	*Rhododendron* sp.	China	No information available	Absent	[150]
83	*H. elasticae*	-	-	No information available	Absent	[151]
84	*H. endiandrae*	On leaves of *Endiandra introrsa*/Lauraceae	Australia/New South Wales, Nightcap National Park	*Conidiophores* 200–300 × 5–7 µm, solitary, erect, subcylindrical, straight to flexuous, unbranched, thick-walled, base bulbous, lacking rhizoids, brown, 8–16 septate; *Conidia* (35–)37–45(–57) × (7–)8(–9) µm, solitary or in short chains (2–3), obclavate, thick-walled, finely roughened, brown, 3(–4)-distoseptate	Present	[21,59]
85	*H. eragrostiellae*	On inflorescence and leaves of *Eragrostis bifida*/Poaceae	India/Uttar Pradesh	No information available	Absent	[108]
86	*H. erythrinae*	On leaves of *Erythrina suberosa*/Leguminosae	India/Karnataka	*Conidiophores* 32–42 × 4–5 μm, simple, brownish-yellow; *Conidia* 39–62 μm at base, straight or vermiform, rounded at the apex and flat at the base, pale cinnamon-brown, 4–8-distoseptate	Absent	[91]
87	*H. erythrinicola*	On leaves of *Erythrina humeana*/Fabaceae	South Africa/Eastern Cape	*Conidiophores* 500–1200 × 6–10 mm, fasciculate, subcylindrical, unbranched, brown, becoming pale brown at apex, multiseptate; *Conidia* (70–)80–90(–110) × (9–)10–11(–12) mm, obclavate, straight to curved, apex subobtuse, smooth, medium brown, (6–)7–8(–12)-distoseptate	Present	[22]
88	*H. exasperatum*	On *Dianthus barbatus*/ Caryophyllaceae	UK/Great Britain	*Conidiophores* flexuous, knotted above, each knot bearing oblong conidia; *Conidia* 30–45 × 10–12 μm	Absent	[152]
89	*H. feijoae*	On leaves of *Acca sellowiana*/Myrtaceae (syn: *Feijoa sellowiana*)	North America/Hispaniola island	No information available	Absent	[153]
90	*H. ferrugineum*	On leaves of *Hiraea* sp. and *Heteropterys* sp./Malpighiaceae	U.S.A./San Francisco	*Conidiophores* 8–9 μm thick, filiform, yellow, septate; *Conidia* 50–62 × 11–14 μm, obclavate, subhyaline, last 2 septate hyaline-yellow to yellow	Absent	[141]
91	*H. fici*	On leaves of *Ficus retusa*/Moraceae	Philippines, Thailand	*Conidiophores* fusciculate, long, nodulosis, septate; *Conidia* 18–20 × 5–6 μm, cylindrical, reddish-brown, 3-distoseptate	Absent	[137,154]
92	*H. ficinum*	On leaves of *Ficus ulmifolia*/Moraceae	Philippines	*Conidiophores* 250 × 6 μm, filiform, septate; *Conidia* 50–60 × 6–8 μm, obclavate, 4–5-distoseptate	Absent	[137]
93	*H. filicicola*	On leaves of *Lygodium* sp./Lygodiaceae and of *Selaginella* sp./Selaginellaceae	Peru	*Conidiophores* 400 × 3–5 μm thick, erect, simple, filiform, septate; *Conidia* 30–40 × 6–10 μm, cylindrical-fusoid or clavate, both side blunt, 3–5-distoseptate	Absent	[155]
94	*H. flagellatum*	On mycelium of *Meliola*, in leaves of *Ardisia disticha*/Myrsinaceae	Philippines	*Conidiophores* 2.5–4 μm thick, erect, sub-hylaline	Absent	[156]
95	*H. flumeanum*	On leaves of *Bambusa* sp./Bambuseae	Philippines	*Conidiophores* 90–100 × 6–7 μm, dense, fasticulate, filiform; *Conidia* 35–40 × 9–12 μm, obclavate, 3-distoseptate	Absent	[157]
96	*H. fumagineum*	On leaves of *ficusulmifolia*/ Moraceae	Philippines	*Conidiophores* 240–300 × 7 μm, filiform, septate; *Conidia* 35 × 9–10 μm, oblong-obclavate, 3-distoseptate	Absent	[137]
97	*H. gibberosporum*	*Musa cavendishii*/Musaceae	Somalia	No information available	Absent Present	[158]
98	*H. glabroides*	On *Meliola glabroides*, on *Piper aduncum*/ Piperaceae	Puerto Rico	*Conidiophores* 100–140 × 7 μm; *Conidia* 40–81 × 6–7 μm, 3–6-distoseptate	Absent	[159]
99	*H. gleicheniae*	On leaves of *Dicranopteris linearis* (=*Gleichenia dichotoma*)/ Gleicheniaceae	U.S.A./Hawaii Islands	No information available	Absent	[123]
100	*H. gossypii*	On living leaves and bracts of *Gossypium* sp./ Malvaceae	North America	*Conidiophores* 40–185 × 6.5–8.5 μm, singly or in groups of three to six, straight cylindrical to nodose or bent, brown, 5 septate; *Conidia* 35–118 × 11.7–18.4 μm, elliptical, curved, rarely straight, light to dark fuliginous, thick walled, rounded at the ends, 1–8-distoseptate	Absent	[160]
101	*H. grewiae*	On leaves of *Grewia* sp./Malvaceae	Democratic Republic of the Congo	*Conidiophores* 80–120 × 5–8 μm, fasciculate, septate; *Conidia* 35–45 × 8–10 μm, fusoid, 2–4-distoseptate	Absent	[161]
102	*H. guangxiense*	On dead branches of unidentified tree	China/Guangxi, Shanglin	*Conidiophores* 330–850 μm long, 15–20 μm wide just above the base and 8–13 μmwide toward the apex, fasciculate, simple, straight or flexuous, sub-cylindrical, thick-walled, smooth, brown, with 1–3 well-defined small pores at the apex, 1–4 septate; *Conidia* 76–110 μm long, 16–22 μm wide in the widest part, narrowing towards the apex to 3–6μm wide, straight or curved, obclavate, smooth, middle brown, paler towards the apex, 9–17-distoseptate, with a large dark blackish-brown scar at the base, 1.5–3.5 μm thick	Absent	[128]
103	*H. guianense*	*Meliola guianensis* parasitic on mycelium on living leaves of *Theobroma cacao*/ Malvaceae	Guyana	No information available	Absent	[162]
104	*H. heringerianum*	*Tipuana speciosa*/ Fabaceae	Brazil	No information available	Absent	[163]
105	*H. hispanicum*	On dead corticated twigs of *Juglans regia*/Juglandaceae	Asturias, Selviella, Spain	*Conidiophores* 130–540 μm long, 13–22.5 μm wide at the base, tapering to 8–15 μm near the apex, solitarily or in small groups, erect, simple, straight or flexuous, thick-walled, subcylindrical, smooth, dark to blackish brown, paler near the apex, with well-defined small pores at the apex, 1–2 septate; *Conidia* 69–99(–130) × (17–)18–21(–24) μm, obclavate, straight or flexuous, thin-walled, smooth, pale brown, (4–)6–11(–14)-distoseptate, with a blackish-brown 4–6 μm wide scar at the base	Present	[21]
106	*H. hispaniolae*	On living leaves of *Manihot utilissima*/ Euphorbiaceae	Dominican Republic/Haiti	*Conidiophores* sub-hyaline to light-grey, when old, with an almost hyaline tip; *Conidia* 14.8–(53.5–)81.4 × 7.4–(11–)14.8 μm, sub-hyaline to smoky, irregular, cylindric-elongate to ellipsoidal, straight or slightly curved, with the basal end applanate, 1–8-distoseptate	Absent	[112]
107	*H. hunanense*	On dead branches of unidentified tree	China/Zhangjiajie, Hunan	*Conidiophores* 70–226 × 5–7 above, 8.5–14 μm base, solitary or fasciculate, simple, cylindrical, straight or flexuous, thick-walled, smooth, brown, well-defined small pores at the apex, 1–3 septate; *Conidia* 56–127 × 10–14 base, apex 2–4 μm, obclavate, straight or curved, smooth, middle brown, paler towards the apex, 4–12-distoseptate, blackish-brown scar at the base, 1.5 μm thick	Absent	[67]
108	*H. hygrophilae*	On leaves of *Hygrophila brasiliensis*/Acanthaceae	Dominican Republic	No information available	Absent	[89]
109	*H. insigne*	On leaves of *Mallotus philippensis*/ Euphorbiaceae	Philippines	*Conidiophores* 600–800 × 50 μm, fasciculate, filiform, blackish, septate; *Conidia* 45–55 × 7–8 μm, obclavate, often curved, 4–5-distoseptate	Absent	[137]
110	*H. insuetum*	On living leaves of *Philodendron sodiroi* (*=Piplocarpha sodiroi*)/Araceae	Ecuador/Pichincha	*Conidiophores* 2.5–5 μm thick, olive brown or dark brown; *Conidia* 17–38 × 7–12 μm, oblong, ellipsoid or oblong-ellipsoid fusiform and often subclavate, rarely cylindrical, often straigtly, rarely curved, olive brown or dark-brown, (3–)5–7(–9)-distoseptate, scared or a little more often in the middle constricted	Absent	[122]
111	*H. ipomoeae*	On leaves of *Ipomoea reptans*/ Convolvulaceae	China/Taiwan	No information available	Absent	[130]
112	*H. iranicum*	On living leaves of *Indigofera* sp./Fabaceae	Iran/Bandar Abbas	*Conidiophores* 40–75 × 6-9 μm, dense, curved, rarely straight, dark-brown, septate; *Conidia* 36(–42) × 7–11 μm, oblong, narrowly ellipsoid or curved, obtuse at both ends, straight or curved, sometimes irregular, olive, 1–3-distoseptate	Absent	[164]
113	*H. italicum*	On dead branch of *Alnus glutinosa*/ Betulaceae	Italy	*Conidiophores* (190–)330–600 × (12–)16–18(−20) μm, aggregated, erect, straight or slightly flexuous, unbranched, cylindrical, dark brown, 13–25 septate; *Conidia* 58–78 × 15–19(−23) μm, obclavate, straight or curved, pale brown to brown, slightly truncate and black at base, rounded, narrowed, 6–11-distoseptate	Absent	[61]
114	*H. juglandinum*	On dead corticated twigs of *Juglans regia*/ Juglandaceae	Austria/Niederösterreich/Gießhübl, Italy	*Conidiophores* (175–)215–325(–455) μm long, 11–23 μm wide at the base, 8.5–14 μm wide near the slightly inflated apex, fasciculate, erect, simple, straight or flexuous, thick-walled, sub-cylindrical, smooth, brown to dark brown, darker to black at the apex, the latter with a well-defined apical pore; *Conidia* (69–)89–145(–205) × (15.0–)16.5–20.0(–25.0) μm, rostrate, straight or flexuous, thin-walled, smooth, pale brown, (5–)9–17(–20)-distoseptate, blackish-brown scar at the base	Present	[21]
115	*H. juglandis*	*Juglans* sp./ Juglandaceae	China, Yunnan	No information available	Absent	[165]
116	*H. kakamegense*	On dead attached twig of *Uvariopsis congensis*/ Annonaceae	Kenya	*Conidiophores* 250–550 × 8–12 μm, solitary, unbranched; *Conidia* 30–90 × 8–10 μm, in the broadest part, uniformly tapering to 2–4 μm wide at at the apex, solitary, simple straight or somewhat curved, obclavate, rostrate, subhyaline, smooth, 4–15-distoseptate	Absent	[166]
117	*H. kalakadense*	On dead unidentified twig	India/Tamil Nadu	*Conidia* 13–15 μm	Absent	[21]
118	*H. kalopanacis*	On dead wood of *Kalopanax septemlobus*/ Araliaceae	Russia/Primorye	No information available	Absent	[167]
119	*H. kok-saghyz*	In seeds of *Taraxacum kok-saghyz*/Asteraceae	Russia	No information available	Absent	[168]
120	*H. kyllingae*	*Kyllinga* sp./ Cyperaceae	Uganda	No information available	Absent	[169]
121	*H. lablab*	On leaves of *Dolichos lablab*/Fabaceae	China/Taiwan	No information available	Absent	[130]
122	*H. leucadendri*	On leaves of *Leucadendron* sp./ Proteaceae	South Africa/Western Cape Province, Helderberg Nature Reserve	On MEA and PDA *Conidiophores* 100–300 × 4–6(–7) μm, erect, subcylindrical, thick-walled, medium brown, multiseptate; *Conidia* (35–)70–110(–170) × (6–)7–8(–11) μm, obclavate to subcylindrical, straight to slightly curved, thick-walled, medium brown, (3–)4–6(–10)-distoseptate	Present	[21]
123	*H. leucosykes*	On *Meliola*, on leaves of *Leucosyke capitellata*/ Urticaceae	Philippines	*Conidiophores* 300 × 7–8 μm, erect, brown, septate; *Conidia* 30 × 8 μm, 3-distoseptate	Absent	[156]
124	*H. ligustri*	On dead branches of *Ligustrum quihoui*/ Oleaceae	China/Guangxi, Nanning	*Conidiophores* 127–700 μm long, 9.5–18 μm diam just above the base and 8.5–10 μm diam towards the apex, solitary, simple, straight or flexuous, smooth or verruculose, thickwalled, dark brown, with 1–3 well-defined small pores at the apex, 1–4 septate; *Conidia* 24–38.5 × 9.5–13 μm, obclavate, straight or slightly curved, rostrate or pseudorostrate, smoothwalled, pale brown, subhyaline towards the apex, 4–6-distoseptate, with a large dark blackish-brown scar at the base, 1–2 μm thick	Absent	[128]
125	*H. litseae*	*Litsea polyantha*/ Lauraceae	India/Assam	No information available	Absent	[170]
126	*H. livistonae*	On leaves of *Livistona australis*/Arecaceae	Australia/New South Wales, Murramarang National Park	*Conidiophores* 500 × 4–6 μm, erect, flexuous, cylindrical, smooth to rough-walled, medium brown, multiseptate; *Conidia* (25–)40–55(–65) × (7–)8–9 μm, subcylindrical, straight, smooth, medium brown, apex obutuse, base somewhat obconic, (3–)4–6(–7)-distoseptate	Present	[171]
127	*H. longisinuatum*	Palmae rotten trunk	Peru	*Conidiophores* 20–75 × 3.5–5 μm; *Conidia* 65–220(–1000) × 8–10.5 μm, solitary, long, narrowly obclavate, 9–22-distoseptate	Absent	[109]
128	*H. lonicerae*	On *Lonicera* sp./ Caprifoliaceae	Brazil	No information available	Absent	[111]
129	*H. lophirae*	On leaves of *Lophiraalata* sp./Ochnaceae	Sierra Leone	*Conidiophores* 110–200 × 3–4 μm thick, simple, bluntly rounded ends; *Conidia* solitary 15–29 × 3.5–4.5 μm, oblong or oblong-cylindrical, hook or curved, smooth, olive- brown, 1–2 guttulate, 1–3-distoseptate	Absent	[172]
130	*H. lunzinense*	No information available	No information available	No information available	Absent	[173]
131	*H. lusitanicum*	On *Alnus glutinosa*/ Betulaceae	Portugal	No information available	Absent	[174]
132	*H. lycopersici*	On *Solanum lycopersicum*/Solanaceae	Guinea	No information available	Absent	[175]
133	*H. machaerii*	On *Machaerium* sp./ Fabaceae	Brazil	No information available	Absent	[111]
134	*H. macilentum*	On rotten wood	UK/Great Britain	*Conidiophores* erect, simple, fusiform, 7–10 septate; *Conidia* 0.5-0.65 × 0.1 mm	Absent	[176]
135	*H. magnisporum*	On dead fallen branches of an unknown woody plant	Japan	*Conidiophores* 150–270 μm long, 9.5–13 μm thick at the apex, 8.5–13.5 μm thick at the base, single or fasciculate, straight or flexuous, smooth walled, brown to dark brown, sometimes paler toward the apex, septate; *Conidia* 100–203 × 12.5–22.5 μm tapering gradually to 2.5–5 μm thick near the apex, solitary, obclavate or rostrate, straight or flexuous, pale olive-brown to pale brown, paler toward the apex, 7–18-distoseptate, with a blackish-brown to black scar, 4–7 μm thick	Present	[177,178]
136	*H. makilingense*	On dead branches of *Paramignya monophylla*/ Rutaceae	Philippines	*Conidiophores* 400–600 × 7–9 μm, dense, erect, curved, brown, septate; *Conidia* 100–300 × 10–12 μm, obclavate, 12–18-distoseptate	Absent	[179]
137	*H. manihotis*	on living leaves of *Manihot* sp./ Euphorbiaceae	Brazil	*Conidiophores* 50–95 × 4–6 μm, 4–6 septate; *Conidia* 40–50 × 6–8 μm, vermiform, clavate to subfusoid, olives, 4–7-distoseptate	Absent	[180]
138	*H. marantae*	On leaves of *Maranta arundinacea*/ Marantaceae	China/Taiwan	No information available	Absent	[130]
139	*H. massarinum*	*Berchemia racemose*/ Rhamnaceae	Japan	*Conidiophores* 380–810 × 7–9 wide at the apex, 13.5–21 wide at the base μm, 15–25 septate; *Conidia* 17–56.5 × 5–9 μm, tretic, solitary or in short chains (5–6), obclavate, rostrate, pale brown, smooth, with or without guttules, 1–8-distoseptate	Present	[19]
140	*H. mattiroloi*	On branches of *Sideroxylon oxyacantha*/ Sapotaceae	Etiopia	No information available	Absent	[181]
141	*H. mayaguezense*	On culms and leaves of *Paspalum conjugatum*/ Poaceae	Puerto Rico	*Conidiophores* 300–500 × 18–22 μm; *Conidia* 135–155 × 35–45 μm, fusoid to clavate, 3–4-distoseptate	Absent	[182]
142	*H. melastomacearum*	On *Meliolamelastomacearum*, on *Miconiaracemose*/ Melastomataceae	Puerto Rico	*Conidiophores* 280 × 3 μm; *Conidia* 14–21 × 3.5–6 μm, ellipsoid, 3-distoseptate	Absent	[159]
143	*H. meliae*	On leaves of *Melia azedarach*/Meliaceae	Dominican Republic	*Conidiophores* 250–350 × 15–22 μm, simple, aggregated, branched, olive-brown to black, septate; *Conidia* 70–100 × 12–15 μm, elongate, fusoid, or clavate	Absent	[183]
144	*H. melioloides*	On leaves of *Uvaria* sp./Annonaceae	Philippines	*Conidiophores* 250–300 × 6–8 μm; *Conidia* 35–45 × 9–10 μm, obclavate, 3-distoseptate	Absent	[137]
145	*H. microsorum*	On twigs of *Quercus ilex*/ Fagaceae	England, Italy	*Conidiophores* 100–550 × 8–14 μm, fasciculate, simple, flexuous, cylindrical, smooth-walled, dark brown, with a pore at the apex and often 1–2, septate; *Conidia* 60–(114–)160 × 12–(17–)22 μm thick in broadest part, tapering to 4–10 μm near the apex, obclavate, smooth-walled, pale to mid golden-brown, 9–17-distoseptate, with 5–7 μm wide at the scar	Present	[184]
146	*H. microsporum*	From soil	India/Maharashtra	*Conidiophores* 234–468 × 10.8 μm, pale brown, 10–16 septate; *Conidia* 26–41 × 22 μm, fusoid, widest at the middle, brown, 2–7-distoseptate	Absent	[18]
147	*H. minimum*	On dead decorticatd branches	UK/Great Britain/England	*Conidiophores* erect, simple, septate; *Conidia* 12–14 × 3–4 μm, fusiform, obtuse at the ends, triseptate, scarcely constricted, hyaline	Absent	[185]
148	*H. multiseptatum*	On dead branches	China/Guangdong	*Conidiophores* 390–650 × 10–14 μm wide at the base, 7–9 μm at apex, simple, subcylindrical, straight or slightly flexuous, smooth-walled, brown to dark brown, paler towards the apex, with 1–3 well definded, small pores, 1–3 septate; *Conidia* 78–190 μm long, 11–16 μm thick in the widest part, narrowing toward the apex to 3–6 μm, tretic, straight or slightly flexuous, obclavate or whip-like, smooth-walled, pale brown paler toward the apex, 13–25-distoseptate, with a dark blackish-brown scar at the base	Absent	[129]
149	*H. nadsonii*	On fibers of *Gossypium* sp./ Malvaceae	Russia	No information available	Absent	[186]
150	*H. nanjingense*	On dead branches of an unidentified tree	China/Jiangsu, Nanjing	*Conidiophores* 250–470 × 6.9–7.7 μm, solitary or fasciculate, simple, straight or flexuous, thick-walled, sub-cylindrical, smooth, brown to dark brown, with well-defined small pores at the apex,1–4 septate; *Conidia* 64.5–170.5 μm long, 7.3–10.3 μm wide in the widest part, narrowing towards the apex to 5.0–6.8 μm wide, subulate or nearly whip-like, straight or curved, thin-walled, smooth, pale brown, 6–17-distoseptate, with a blackish-brown scar at the base, 1.4–2.7 μm thick	Present	[187]
151	*H. naviculare*	On leaves of *Euphorbia* sp./ Euphorbiaceae	Brazil/Tubarão	*Conidiophores* 6–8 μm thick, branched, often curved, yellow, septate; *Conidia* 50–84 × 11–16 μmnaviculiform, hyaline at length, very pale with brown	Absent	[188]
152	*H. naviculatum*	On dead herbaceous stems of *Solidago* sp./ Asteraceae	U.S.A./New York	No information available	Absent	[189]
153	*H. newbouldiae*	On leaves of *Newbouldialaevis*/ Bignoniaceae	Guinea	No information available	Absent	[190]
154	*H. novae-zelandiae*	On dead wood and bark of *Vitex lucens*/ Lamiaceae	New Zealand	*Conidiophores* 165 μm long, 4.8–7(–9) μm, erect, single or in groups, simple, or once-branched at the base, straight or flexuous, subcylindrical, brown to dark brown below, very pale brown to subhyaline above, 15 septate; *Conidia* 13.5–16.2 × 7.2–9.0 μm, solitary, obovoid, sometimes slightly, smooth, the 2 lower cells being brown and the distal cell paler with a dark band of wall overlying each septum, 2-distoseptate	Absent	[191]
155	*H. obpyriforme*	On dead branches of unidentified tree	China/Guangxi	*Conidiophores* 225–460 μm long, 9.5–13 μm diam just above the base and 6–8.5 μm diam towards the apex, arising singly from the upper cells of the stromata, simple, subcylindrical, straight or flexuous, dark brown, paler towards the apex, with well-defined small pores at the apex,1–3 septate; *Conidia* 47–74 μm long, 14–19 μm diam in the widest part, narrowing in diameter towards the apex to 2.5–5 μm, straight or slightly curved, obpyriform, smooth-walled, middle brown, paler towards the apex, 5–9-distoseptate, with a large dark blackish-brown scar at conidium base, 1–2 μm thick	Absent	[128]
156	*H. ocoteae*	On *Meliola ocoteae*, on *Guareatrichilioides*	Puerto Rico	*Conidiphores* 135–200 × 4 μm, septate; *Conidia* 20–28 × 4–6 μm, 3-distoseptate	Absent	[159]
157	*H. oligosporum*	Holotype of *Sporidesmium olivaceum*: on rotten branches of *Tilia* sp. Lectotype of *Coryneumoligosporum*, here designated: on rotten branches of *Corylus* sp. Epitype of *Sporidesmiumolivaceum* and of *Coryneumoligosporum*: on dead corticated twigs of *Tiliacordata* sp.	Austria, Czech Republic, Germany	From Epitype specimen [21] *Conidiophores* (17–)22–35(–46) × (8.0–)8.5–10.5(–11.5) μm, densely crowded, erect, simple, straight, cylindrical to slightly swollen at the apex, brown to dark brown, darker at the apex, smooth, 0–2 septate; *Conidia* (37–)59–80(–124) × (14.8–)15.8–18.0(–20.0) μm, tapering to 4–10.5 μm at the distal end, with 4–8 μm wide, dark brown to black scar at the base, obclavate, sometimes rostrate, straight or curved, smooth but occasionally wrinkled with age, pale brown to brown, paler toward the apex, 6–12(–16)-distoseptate	Present	[21,124]
158	*H. olisipponense*	Culture from the perithecia stage of *Pyrenophora polytricha*	-	No information available	Absent	[192]
159	*H. oplismeni*	On leaves of *Oplismenus cotnpositus*/Poaceae	China/Taiwan	No information available	Absent	[130]
160	*H. orchidacearum*	On leaves of *Neottia ovata* (=*Listera ovata*)/Orchidaceae	France	No information available	Absent	[193]
161	*H. orthospermum*	On rotten wood	U.S.A./New York	*Conidiophores* 50–60 × 5 μm, erect, simple, fasciculate, straight, dark, 3–4 septate; *Conidia* 60–80(–110) × 10–12 μm, cylindrical, straight, apex rounded, tuncated at base, 12–14-distoseptate	Absent	[194]
162	*H. oryzae-microsporae*	On *Oryza sativa*/Poaceae	Japan	No information available	Absent	[195]
163	*H. ovoideum*	On dead branches of tree	China/Jilin	*Conidiophores* 380–510 × 15–25 μm diam just above the base, 7.5–10 μm diam towards the apex, arising singly from the upper cells of the stromata, simple, subcylindrical, straight or flexuous, thick-walled, smooth, brown to dark brown, paler towards the apex, with 1–3 well-defined small pores at the apex, 1–6 septate; *Conidia* 27–61 × 13–21 μm diam in the widest part, narrowing towards the apex to 4.5–8.5 μm, straight, ovoid, to ellipsoidal, smooth-walled, moderately brown, paler towards the apex, 3–8-distoseptate, with a large dark blackish-brown scar at the base, 1.5–2.5 μm thick	Absent	[128]
164	*H. pachystelae*	On living leaves of *Synsepalumm solo*(=*Pachystelam solo*)/ Sapotaceae	Tanzania	*Conidiophores* 300–350 × 6–8 μm, erect, simple, septate; *Conidia* 35–50 × 10–13 μm, fusoid or oblong clavate or lanceolate, 3-5-distoseptate	Absent	[196]
165	*H. palaestinum*	On stems and flowers of *Dianthus* sp./ Caryophyllaceae	Israel	*Conidiophores* 30–160 × 6–8 μm, fasciculate, 8–16 aggregate, simple, bent, thick-walled, coffin terminal obtuse, thin, yellow or colorless, 5–7 septate; *Conidia* 60–120 × 9–12 μm, solitary, obclavate, rectiusculis or curved, pale-olive, minute-granule, thick-walled, towards colorless above, 5–7-distoseptate	Absent	[197]
166	*H. palmigenum*	On rotten fruit of *Cocos nucifera*/Arecaceae; On petriole and rachis from reference specimen	Brasil/Pará, Papua New Guinea	From reference specimen [190]; *Conidiophores* 132.5–195 × 5–6 μm, solitary, erect, simple, cylindrical, straight or flexuous, smooth, brown, light brown at the apex, 7–10 septate; *Conidia* 38–53 × 8–11 base, 3–4 μm apex, solitary, in small chains, obclavate or cylindrical, straight or slightly curved, simple, smooth, brown with light brown at apical cell, 6–10-distoseptate	Absent	[198,199]
167	*H. panici*	On leaves of *Panicum maximum*/Poaceae	Indonesia/Java	*Conidiophores* 115–180 × 8–10 μm; *Conidia* (35–)50–75 × (7–)10–13 μm, ellipsoidal-truncate, ellipsoidal-elongate, dull-brown, (1–)3(–4)-distoseptate	Absent	[159]
168	*H. papulosum*	On bark of *Malus sylvestris* or *Pyrus communis*/Rosaceae	West Virginia	No information available	Absent	[200]
169	*H. parathesicola*	On *Meliola parathesicola*, on *Parathesis serrulate*/Primulaceae	Puerto Rico	*Conidiophores* 120 × 4 μm, solitary; *Conidia* 17–20 × 4–6 μm, base truncate, apex beaked, beak often 7 μm long, 1–3-distoseptate	Absent	[159]
170	*H. paulense*	On leaves of Myrtaceae	Brazil/São Paulo	*Conidiophores* 3–4.5 μm thick, brown, septate; *Conidia* 15–24 × 4 μm, fusoid, brown, 3-distoseptate	Absent	[115]
171	*H. penniseti*	On leaves of *Pennisetum glaucum* (=*Pennisetum typhoides*)/Poaceae	India/Uttar Pradesh	No information available	Absent	[108]
172	*H. philippinum*	On decaying leaves of *Arenga mindorensis*/ Arecaceae	Philippines	*Conidiophores* 300–400 × 6–7 μm, fasciculate, filiform, curved, septate; *Conidia* 33–35 × 8–9 μm, obclavate, 4-distoseptate	Absent	[137]
173	*H. philodendri*	On *Meliola philodendri*, on *Philodendron krebsii*/Araceae	Puerto Rico	*Conidiphores* 400 × 3–4 μm; *Conidia* 24–35 × 5–9 μm, clavate, 3-distoseptate	Absent	[159]
174	*H. phomatae*	On bark of *Acer pennsylvanicum*/ Sapindaceae	U.S.A./New York	No information available	Absent	[189]
175	*H. phyllantheum*	On dead branches hanging down of *Phyllanthus* sp./Phyllanthaceae	Philippines	*Conidiophores* 180–200 × 4.7–6 μm, fillifrom, blackedned, septate; *Conidia* 80–90 × 9–10 μm, obclavate, long, 9–11-distoseptate	Absent	[137]
176	*H. piperis*	On leaves of *Piper betle*/Piperaceae	China/Taiwan	No information available	Absent	[130]
177	*H. portoricense*	On dead branches hanging down of *Phyllanthus* sp./Phyllanthaceae	Philippines	*Conidiophores* 25–250 × 2–5 μm; *Conidia* 30–60 × 6–10 μm, elongate-fusoid, olive-brown or brown, (2–)4-distoseptate	Absent	[86,201]
178	*H. proliferatum*	On grain of *Triticum* sp./Poaceae	India/Maharashtra	Colony on PDA; *Conidiophores* 292–510 × 7–13.8 μm, unbranched, pale, olivaceous, 5–20 septate; *Conidia* 23–126 × 11.5–13.8 μm; cylindrical, olivaceous, 3–13-distoseptate	Absent	[101]
179	*H. pseudomicrosorum*	On dead branches of unidentified tree	China/Changbaishan, Jilin	*Conidiophores* 155–288 × 11–15 μm, fasciculate, simple, cylindrical, straight or flexuous, smooth, dark brown, paler towards the apex, with 1–3 well-defined small, 1–4 septate; *Conidia* 82–142 × 17–27 μm in the widest part, narrowing towards the apex to 3–6 μm diam, tretic, straight or slightly flexuous, obclavate, smooth-walled, brown, paler towards the apex, 7–16-distoseptate, with a large dark blackish-brown scar at the base, 2–4 μm thick	Absent	[128]
180	*H. pseudotsugae*	On bark and resin exudations of *Pseudotsuga taxifolia var. glauca*/Pinaceae	U.S.A.	*Conidiophores* scattered on aerial hyphae with usually one at each cell; *Conidia* 65–105 × 14–15 µm, opaque, black or greenish black, smooth walls, with 8–14-distoseptate	Absent	[202]
181	*H. purpurascens*	On leaves of *Panicum purpurascens*/Poaceae	U.S.A./Florida	No information available	Absent	[203]
182	*H. pyracanthae*	*Pyracantha* sp./Rosaceae	Portugal	No information available	Absent	[204]
183	*H. quercicola*	On dead corticated branches of *Quercus* cf. *reticulata*/Fagaceae	U.S.A.	*Conidiophores* (115–)133–226(–300) μm long, 14–20 μm wide at the base, tapering to 10–15 μm near the apex, solitarily or fasciculate, simple, straight or flexuous, cylindrical, thick-walled, smooth, brown to dark brown, with well-defined small pores at the apex; *Conidia* 60–100 × 15–22 μm, straight or flexuous, obclavate, smooth-walled, brown, 8–10-distoseptate, with blackish-brown to black scar at the base	Absent	[21]
184	*H. quercinum*	On dead corticated twigs of *Quercus petraea*/Fagaceae	Austria/Niederösterreich, Spitzerberg	*Conidiophores* (40–)74–199(–332) μm long, 11–18 μm wide at the base, tapering to 8.5–13.5 μm near the apex, solitarily or fasciculate, simple, straight or flexuous, cylindrical, smooth, brown to dark brown, with well-defined small pores at the apex, 1–5 septa; *Conidia* (47–)78–130(–201) × (13.2–)15.3–18.0(–20.5) μm, straight or flexuous, rostrate, smooth-walled, brown, 8–13(–20)-distoseptate, with blackish-brown to black scar at the base	Present	[21]
185	*H. repens*	On bark of dead *Acer grandidentatum*/ Sapindaceae	U.S.A./Utah, Red Butte Canyon	*Conidia* 40–45(–60) × 8–9 μm, sub-oblong, 5–12-distosepate	Absent	[205]
186	*H. reyesii*	On dead branch of *Guioa* sp./ Sapindaceae	Philippines	*Conidiophores* 130 × 8–10 μm, erect, brown, septate; *Conidia* 34–112 × 8–13 μm, tereti-fusoid, brown, ends hyaline, 5–14-distosepate	Absent	[137]
187	*H. rhodomyrti*	On leaves of *Rhodomyrtus tomentosa*/Myrtaceae	China/Guangdong	*Conidia* 42–60 × 17–20 μm, fusoid, brown, 5–7-distosepate	Absent	[206]
188	*H. rhopaloides*	On decraying stem of *Brassica oleracea*/Brassicaceae	Britain, France, Germany, Italy, Portugal	*Conidiophores* short, dark brown-black, 12–14 septate; *Conidia* 0.04–0.1 mm long, 0.08 mm wide, straight or slightly curved	Absent	[207,208]
189	*H. schelkownikowii*	On branches	Armenia, Azerbaijan, Georgia, Russia	No information available	Absent	[209]
190	*H. scolecoides*	On dry woood	Germany/Reichenberg	*Conidiophores* simple, branched; *Conidia* 80 × 7.5 μm, torulus, fusciculate, septate, yellow	Absent	[96]
191	*H. sechiicola*	On *Sechium edule*/ Cucurbitaceae	Puerto Rico	No information available	Absent Present	[210]
192	*H. sichuanense*	On dead branches of tree	China/Sichuan	No information available	Absent Present	[211]
193	*H. solani*	On stem of *Solanum nigrum*/Solanaceae (type); *Citrus linella*; *Leucaena glauac*; *Solanum dulcamara*; *S. nigrum*; *Solanum tuberosum*	England, Guernsey, New Zealand, New Guinea, Sierra Leone, Wales	*Conidiophores* 120–600 × 9–15 μm thick near base, 6–9 μm thick near the apex, erect, simple, straight or flexuous, smooth or occasionally, brown to very dark brown, paler near apex, septate, with small pores at apex, 1–8 septate; *Conidia* (24–)39–85 × (7–)9–11 μm, straight or curved, obclavated, smooth-walled, subhyaline to brown, 2–8-distosepate, with a welll-defind dark brown to black scar at base	Present	[212]
194	*H. solitarium*	On leaves of *Iris* sp./Iridaceae	U.S.A./Minnesota	*Conidiophores* 60–150 × 6 μm, solitary, slightly fasciculate, erect, swollen at the base, lighter colored at the apex, dark brown, septate; *Conidia* 24–30 × 8–9 μm, oblong-elliptical, sometimes slightly curved, dark brown, at first 2–4 guttulate, 3–5-distosepate	Absent	[213]
195	*H. spirotrichum*	On withered leaves of *Cyrtophyllum fragrans*/Gentianaceae	Singapore	*Conidiophores* 190–220 × 6 μm, fasciculate, filiform, brown, septate; *Conidia* 23–25 × 9 μm, oblong-obclavate, gently curving, brown, 3-distosepate	Absent	[214]
196	*H. spurirostrum*	On dead branches of tree	China/Sichuan	No information available	Absent	[211]
197	*H. subapiculatum*	On dead wood of *Sambucus callicarpa*/Adoxaceae	U.S.A./Washington	*Conidiophores* 8–10 μm thick; *Conidia* 35–80 × 12–16 μm, oblong or subfusiform, 6–7-distosepate	Absent	[215]
198	*H. subhyalinum*	On living leaves of *Phoenix hanceana*/Arecaceae	China/Guangdong	*Conidiophores* 120–200 × 10–12 (basal), above 6–8.5 μm thick, simple or fasciculate, erect, subcylindrical, brown, pores 1–3 μm, septate; *Conidia* 72–125 × 9–11.5 μm, obclavate, straight or flexuous, subhyaline, apex 2.5–5 μm, black at tip, 6–9-distosepate, dark blackish-brown scar	Absent	[129]
199	*H. submersum*	On submerged decaying wood	China/Yunnan	*Conidiophores* 239–423 × 8.5–15.5 μm, solitary or in group of 2–4, unbranched, straight or curved, smooth, dark brown, paler towards to the apex, bulbous at base 9–14 septate; *Conidia* 41–55 × 14.5–18.5 μm, straight or curved, wider below than apex, truncate and dark at base, apically rostrate and pale, smooth, pale brown to mid-brown, guttulate, 6–10-distosepate	Present	[24]
200	*H. subsimile*	On withered and dead leaves of *Bruguiera sexangula* (=*Bruguiera eriopetala*)/Rhizophoraceae	Singapore	*Conidiophores* 200–250 × 8–9 μm, filiform-fasciculate, brown, septate; *Conidia* (38–)45–50 × 11–12(–14) μm, brown, 3-distoseptate	Absent	[216]
201	*H. syzygii*	On bark canker of *Syzygium* sp./Myrtaceae	South Africa/Eastern Cape Province	*Conidiophores* 150–400 × 10–15 mm, fasciculate, unbranched, clavate at apex, dark brown, multiseptate; *Conidia* (70–)80–100(–150) × (19–)22–23(–25) mm, obclavate, curved, apex subobtuse, warty, inner surface striate, medium brown, (7–)9–12-distoseptate	Present	[22]
202	*H. theobromae*	On leaves of *Theobroma cacao*/Malvaceae	Italy	*Conidiophores* 500–1000 μm, erect, 6–10 septate; *Conidia* 60–160 × 12–20 μm, obclavate to tereti-obclavate	Absent	[217]
203	*H. theobromicola*	On rotten leaves of *Theobroma cacao*/Malvaceae	Dominican Republic/Moca	*Conidiophores* 20–33 × 3.5–5 μm olives-brown, septate; *Conidia* 46–58 × 10–13.5 μm, elliptic-oblong or subfusoid, irregular, 3–5-distoseptate	Absent	[218]
204	*H. tritici*	On seedhead of *Triticum vulgare*/Poaceae	Tanzania	*Conidiophores* 3.5–5 μm, thick fasciculate, erect, sepate; *Conidia* 12–25 × 4–7 μm, subcylindrical-oblong, clavate or fusoid, 2–4-distoseptate, constrict at septum	Absent	[219]
205	*H. tritikernelis*	On kernels of *Triticum aestivum*/Poaceae	India/Bihar	No information available	Absent	[108]
206	*H. turbinatum*	On unidentified wood	Great Britain	*Conidiophores* simple, slender; *Conidia* elongated, turbinatis, tuncatus, apiculate, brown, 4–7-distoseptate	Absent	[220]
207	*H. ubangiense*	On leaves of *Coffea* sp./Rubiaceae	Democratic Republic of the Congo/Ubangi River	*Conidiophores* (2–)3–6 μm, fasciculate, erect, branched, septate; *Conidia* 30–60 × 5–8 μm, fusoid, 3–4-distoseptate	Absent	[221]
208	*H. ustilaginoideum*	On flowers of *Panicum spicatum*/Poaceae	Democratic Republic of the Congo	*Conidiophores* 3–3.5 μm thick, fasciculate; *Conidia* 10–50 × 3.5–4.5 μm, cylindrical or subfusoid, blunted, 1–5-distoseptate	Absent	[121]
209	*H. varium*	On decaying leaves of unidentified plants	Brazil/Pernambuco	*Conidiophores* 150–200 × 10–14 μm, erect, unbranched, straight or flexuous, cylindrical, slightly inflated at the apex, smooth, brown, 5–7 septate; *Conidia* 29–58 × 4–7 μm, cylindrical-obclavate, subcylindrical, oblong or navicular, dry, trick-walled, with wall verrucose or verruculose, gray-brown, lumina pale yellow, (0–)1–4-distoseptate	Absent	[222]
210	*H. varroniae*	On leaves of *Varronia* sp./Boraginaceae	Puerto Rico	*Conidiophores* 160–200 × 4 μm; *Conidia* 27–44 × 6–7 μm, 3-distoseptate	Absent	[223]
211	*H. velutinum*	Fagus sylvatica dead corticated twigs/saprobic on decaying wood submerged in stream	Austria, Wien, Döbling, Kahlenberg/China	From reference specimen [20]; *Conidiophores* 530–655 × 16–18 μm, erect or flexuousk, unbranched, dark brown, 17–23 septate; *Conidia* 67–79 × 15–19 μm, single, obclavate, straight or curved, smooth, pale brown to brown, 7–9-distoseptate, rounded at apex, guttulate when young, non-guttulate at maturity	Present	[16,20]
212	*H. viticis*	On leaves of *Vitis* sp./Vitaceae	Brazil/Pará	*Conidiophores* 80 × 2–3 µm, fasciculate, septate; *Conidia* 12–20 × 2.5–3.5 µm cylindrical, 1–3-distoseptate	Absent	[223]
213	*H. wagateae*	On leaves of *Moullava spicata* (=*Wagatea spicata*)/Fabaceae	India/Karnataka	*Conidiophores* 81–125 × 1.5 –2.5 µm, yellowish-brown, multiseptate; *Conidia* 15.5–28 × 3–4 µm, clavate-cylindric, cinnamon-yellow, rounded at both ends, 2–4-distoseptate	Absent	[91]
214	*H. warpuriae*	On stem of *Warpuria clandestina*/Acanthaceae	Great Britain/England	*Conidiophores* 300–500 × 6–8 µm; *Conidia* 115–190 × 12–14 µm, obclavate, 8–11-distoseptate	Absent	[224]
215	*H. xanthosomatis*	On leaves of *Xanthosoma violaceum*/Araceae	Dominican Republic/Moca	*Conidiophores* 35–90 µm long, septate; *Conidia* 185 × 24 µm, fusoid, subfusoid to subclavate, 3–7(-10)-distoseptate	Absent	[225]
216	*H. xylopiifolii*	On *Asterina*, on *Xylopia sericea*/Annonaceae	Brazil/Pernambuco	*Conidiophores* 85–305 × 5.5–8 µm, erect, 3–5 septate; *Conidia* 38–62 × 8–13.5 µm, cylindrical or clavate, 3–6-distoseptate	Absent	[226]

## Data Availability

Not applicable.
